# International variation in child healthcare practices and implications for childhood cancer diagnosis: a scoping review and cross-country clinician survey

**DOI:** 10.1007/s00431-026-07109-9

**Published:** 2026-06-12

**Authors:** Angela Lopez-Cortes, Aisha Al-Mahdy, Zsuzsanna Jakab, Masako Shimato, Laura Botta, Fabio Didonè, Adela Cañete Nieto, Emmanuel Desandes, Lisa L. Hjalgrim, Angela Polanco, Charles A. Stiller, Bernward Zeller, Gemma Gatta, Kathy Pritchard-Jones, The BENCHISTA Project Working Group, Joanne Aitken, Leisa O’Neil, Danny Youlden, Monika Hackl, Ruth Ladenstein, Elizabeth Van Eycken, Nancy Van Damme, Beatriz De Camargo, Marceli De Oliveira Santos, Carlos A Lima, Walmiro Ramos, Lucrecia Aline Cabral Formigosa, Luciana Ferreira dos Santos, Claudina Agnese Casale, Gil Patrus Pena, Juliana Nativio, Cyntia Asturian Laporte, Cristiana Santos de Menezes Miranda, Cristiane Bastos Daniel, Raimunda Nonata de Paulo, Donaldo B. Veneziano, Angela Pontes de Aquino, Paulo Cesar Fernandes de Souza, Rebeca Valentim Leite, Zdravka Valerianova, Dobrin Konstantinov, Sumit Gupta, Jason D. Pole, Jan Stary, Jaroslav Sterba, Lisa L. Hjalgrim, Jeanette Falck Winther, Keiu Paapsi, Brigitte Lacour, Emmanuel Desandes, Jacqueline Clavel, Claire Poulalhon, Meike Ressing, Claudia Trübenbach, Claudia Spix, Eleni T. Petridou, Evdoxia Bouka, Zsuzsanna Jakab, Miklós Garami, Rocco Galasso, Giuseppe Sampietro, Patrizia Piga, Marcella Sessa, Milena M. Maule, Carlotta Sacerdote, Paola Ballotari, Luigino Dal Maso, Antonina Torrisi, Rosalia Ragusa, Luca Boni, Magda Rognomi, Rosalba Amodio, Francesco Cuccaro, Danila Bruno, Antonio G. Russo, Federico Gervasi, Maria L. Gambino, Elisabetta Borciani, Maria L. Michiara, Luciana Mangone, Gianbattista Spagnoli, Stefano Ferretti, Fabio Falcini, Eugenia Spata, Sonia Manasse, Paolo Coccia, Fabrizio Stracci, Daniela Piras, Pasquala Pinna, Francesca Bella, Adele Caldarella, Teresa Intrieri, Tiziana Scuderi, William Mantovani, Manuel Zorzi, Stefano Guzzinati, Deidre Murray, Tomohiro Matsuda, Kayo Nakata, Miriam J. Azzopardi, Tom Børge Johannesen, Aina H. Dahlen, Bernward Zeller, Jerzy Kowalczyk, Anna Raciborska, Ana M. Ferreira, Gabriela Caldas, Mihaela Bucurenci, Daniela Coza, Vesna Zadnik, Arantza López-de-Munain-Marqués, Fernando Alemda-Vich, Noura Jeghalef-ElKaroui, Montse Puigdemonte, Maria Jose Sanchez, Nuria Aragones, David Parra-Blazquez, Maria Dolores Chirlaque, Marcela Guevara, Elena Pardo, Rafael Peris-Bonet, Adela Cañete Nieto, Marià Carulla, Päivi Lähteenmäki, Claudia E. Kuehni, Shelagh M. Redmond, Otto Visser, Henrike Karim-Kos, Sarah Stevens, Lucy Irvine, Charles A. Stiller, Anna Gavin, Deidre Fitzpatrick, Damien Bennett, David S. Morrison, Karen Smith, Dyfed Wyn Huws, Stephanie Smits, Angela Polanco, Giles Greene, Riccardo Capocaccia, Andrea Di Cataldo, Meric Klein

**Affiliations:** 1 UCL Developmental Biology & Cancer Research Department, UCL GOS Institute of Child Health, London, UK; 2 National Childhood Cancer Registry (NCCR), Hungarian Pediatric Oncology Network (HuPON), Budapest, Hungary; 3 Department of Pediatrics, Semmelweis University, Budapest, Hungary; 4 Evaluative Epidemiology Unit, Department of Epidemiology and Data Science, Fondazione IRCCS Istituto Nazionale Dei Tumori, Milan, Italy; 5 Paediatric Oncology and Hematology Unit, Dept. of Pediatrics, Obstetrics and Gynecoloy, Hospital UiP La Fe, Valencia, Spain; 6 Spanish Registry of Childhood Tumours (RETI-SEHOP), University of Valencia, Valencia, Spain; 7 National Registry of Childhood Cancers, CRESS, UMRS 1153, INSERM, Paris-Cité University, Paris, France; 8 National Registry of Childhood Solid Tumors, Institut de Cancérologie de Lorraine, Vandœuvre-Lès-Nancy, France; 9 Department of Paediatrics and Adolescent Medicine, University of Copenhagen, Rigshospitalet, Copenhagen, Denmark; 10 CCLG: The Children & Young People’s Cancer Association, Leicester, UK; 11 National Disease Registration Service, Transformation Directorate, NHS England, London, UK; 12 Division of Paediatrics and Adolescent Medicine, Oslo University Hospital, Oslo, Norway; 13 Department of Statistics and Quantitative Methods, University of Milano-Bicocca, Milan, Italy

**Keywords:** Child healthcare, Childhood cancer, BENCHISTA

## Abstract

Childhood cancer survival and stage at diagnosis vary internationally. Here, we explore differences in child health surveillance and healthcare practices across countries participating in the International Benchmarking of Childhood Cancer Survival by Stage (BENCHISTA) collaboration—with a focus on timely cancer diagnosis. We conducted a scoping review of five databases (MEDLINE, Embase, SCOPUS, Web of Science, and ProQuest Central) for articles published in English or Spanish between 1 Jan 2012 and 15 Oct 2025, describing child health surveillance and acute care pathways, limited to the 29 countries involved in the BENCHISTA collaboration. Two reviewers independently screened abstracts; a third resolved conflicts. In parallel, a semi-structured questionnaire was distributed (to one general practitioner, one paediatrician) in 27 countries who contributed to the BENCHISTA database. We collected standardised information on national child health practices and validated against published national guidance. Of 2,963 articles screened, 33 were included. Three key themes emerged: (1) healthcare-seeking behaviour of families and their interaction with frontline professionals, (2) awareness of alarm symptoms by caregivers and clinicians, and (3) system-level and training-related factors affecting timely diagnosis. Studies highlighted challenges such as low symptom recognition, variation in paediatric training, and limited referral guidance. Questionnaire responses showed variation in the number of routine child health checks with physical examination for children < 5 years (median 10; range 2–21), paediatric training, and access to diagnostic tools.

*Conclusion*: There is substantial international variation in routine child health surveillance and acute illness assessment. These findings will inform interpretation of BENCHISTA data and may guide future strategies to support early cancer diagnosis.

**Table:** 

**What is Known:** • *Childhood cancer survival and stage at diagnosis vary internationally*. • *Early recognition of alarm symptoms is critical but often delayed due to system-level gaps*.	
**What is New:** • *This study combines a scoping literature review and clinician survey across 27 countries, identifying shared barriers to timely diagnosis*. • *It reveals variation in paediatric training of front-line healthcare practitioners, referral pathways, and child health surveillance practices, even among high-income countries*.

**Supplementary Information:**

The online version contains supplementary material available at 10.1007/s00431-026-07109-9

## Introduction

Childhood cancer remains a leading cause of non-communicable disease-related death globally, with over 400,000 children and adolescents (aged 0–19 years) diagnosed each year [1–3]. Marked disparities exist between high-income countries (HICs) and low- and middle-income countries (LMICs) in both incidence and survival [1, 4]. Survival rates vary between HICs and LMICs [5–7]—advances in diagnostics and treatment have raised 5-year survival rates above 80% in HICS [7], while in LMICs, rates remain lower due to delayed or inaccurate diagnosis, limited access to care, and fewer local treatment options [8]. As emphasised by the World Health Organization, childhood cancer data systems are needed to improve the quality of care and inform policy decisions [3].

Accurate and early diagnosis is particularly challenging due to the rarity and clinical heterogeneity of childhood cancers. While cancer registries in many HICs offer population-level survival data [9], few have examined the pathways by which children with cancer access diagnostic services [10, 11]. Child health checks and surveillance could theoretically provide opportunities for earlier detection of serious childhood diseases, including cancer [12, 13].

The International Benchmarking of Childhood Cancer Survival by Stage (BENCHISTA) project is a global collaboration of cancer registries, clinicians, and researchers that is aimed at better understanding survival variation in childhood cancer and at highlighting areas for improvement [14]. Findings from a BENCHISTA population-level analysis showed that the proportions presenting with advanced (or late-stage) disease at diagnosis varied significantly between some geographical areas for several childhood solid tumours, highlighting the need for earlier diagnosis [15]. Here, through a scoping review, we aimed to map international variation in child health surveillance and acute care practices relevant to the early recognition of childhood cancer in countries participating in the BENCHISTA project. In parallel, through a questionnaire completed by clinicians practising in the countries whose cancer registries contributed to the BENCHISTA database, we sought to describe national child health programmes and systems of paediatric care in relation to the early diagnosis of childhood cancers. These findings will be used to aid interpretation of international variation in stage at diagnosis and survival, already demonstrated in the BENCHISTA project [15, 16].

## Materials and methods

### Literature review

We conducted a scoping literature review to identify publications describing child health surveillance systems, acute care access pathways, and training structures relevant to the diagnosis of childhood illness, including cancer. The literature review was restricted to 29 countries (27 HICs; two LMICs) involved in the BENCHISTA project development (Online resource 1 & 5) [14].

Eligible studies were published between Jan 1, 2012, and Oct 15, 2025, in English or Spanish. Five databases (MEDLINE, Embase, SCOPUS, Web of Science, and ProQuest Central) and PROSPERO were searched. This review was conducted according to the Preferred Reporting Items for Systematic Review and Meta-Analyses (PRISMA) statement [17]. The study inclusion and exclusion criteria are presented in Online resource 1. The search strategy was developed using Medical Subject Headings terms, and keywords from relevant literature were identified and discussed with an independent librarian before implementation. Boolean commands were performed to facilitate a clearer approach and reproducibility of the search strategy. Terms including paediatrics or child, diagnosis, cancer, population, and surveillance were used in the search strategy (Online resource 2). In addition to the formal database searches, we conducted backward and forward citation searching (snowballing) of included studies in the PubMed database. This process identified two additional relevant publications that did not appear in the initial search results.

### Data extraction and statistical analysis

Literature identified by the search strategy was imported into the web-based software Covidence, and duplicates were removed. Two independent reviewers (AL-C and AAM) screened the titles and abstracts of each article. All selected papers were read by two reviewers, and data extraction was performed individually. A third independent reviewer, an expert in paediatric oncology (KP-J), resolved the conflicts. We synthesised all data to enable its visualisation from a quantitative and qualitative perspective. When completing full-text screening, reviewers independently appraised each study’s limitations and, therefore, risk of bias.

Throughout the manuscript, we use the following age definitions in line with standard epidemiological practice: children = 0–14 years, teenagers/adolescents = 15–19 years, and young adults = 20–24 years. Some included studies used different age boundaries (for example, 0–15 years or 0–18 years); the age range reported in the source publication is shown in Tables 1 & 2.

For studies reporting time-to-diagnosis or consultation-frequency data, we extracted the measure of central tendency (median or mean) and the accompanying measure of variability (range, standard deviation, interquartile range, or 95% confidence interval) as reported in the source publication. Where more than one measure was reported, we preferred medians and interquartile range or range, reflecting the typically skewed distribution of diagnostic intervals.

### Clinician survey

To provide insights into potential reasons for variations in tumour stage at diagnosis and overall survival for some childhood cancers already documented in the BENCHISTA project, we developed a semi-structured questionnaire. This was reviewed by an expert panel at Nottingham University (Drs Walker, Shanmugavadivel, and Dandapani). Between March 8, 2022, and May 20, 2022, five medical practitioners tested the pilot questionnaire for consistent interpretation of questions and readability in English for non-native speakers. Adjustments were actioned following feedback from the pilot respondents and the Nottingham group.

The final questionnaire was delivered using Opinio software (version 7.19). It comprised 18 questions, covering the respondent’s role and country of practice, national information on mandatory paediatric training practices and professional organisations, information on free and universally provided routine child health surveillance (number of checks, including those involving physical examination and the professionals providing these check-ups), assessment of the acutely unwell child, and whether written details of alarm signs or symptoms are provided to parents, carers, or guardians (Online resource 3). The questionnaire also requested published sources of information for each country for cross-validation. Respondents had the option to remain anonymous. The questionnaire distribution was restricted to the 27 countries that contributed cancer registry data to Phase 1 of the BENCHISTA study (Online resource 1, 5).

Potential respondents were identified through the BENCHISTA Project Working Group (PWG). For each country, the cancer registry representative(s) identified one general practitioner (or equivalent first-contact clinician) and one general paediatrician practising in that country and sent them a standardised email invitation to complete the online questionnaire. Two reminders were sent over the subsequent eight weeks. Where the first nominee declined or did not respond, a replacement was sought through the PWG contact. Responses were not received from clinicians in Austria, Belgium, France, and the Netherlands; for these four countries, national parameters were obtained from published and publicly available online sources.

Responses from each country were reviewed by two members of the research team (AL-C and KP-J), where two respondents from the same country provided discordant answers to a factual question (for example, the number of universally offered routine health checks, or whether a national programme was legally mandatory), the discrepancy was resolved by reference to the publicly available national guidance cited by the respondents or by direct follow-up with the country representative on the BENCHISTA PWG. It was not possible to follow up consistently with the respondents to the clinician survey as it was optional for them to provide their contact email and not all did so. Where respondents’ answers differed from the cross-referenced published source, the published source or information provided by a representative from that country was taken as the primary record.

## Results

**Fig. 1:**
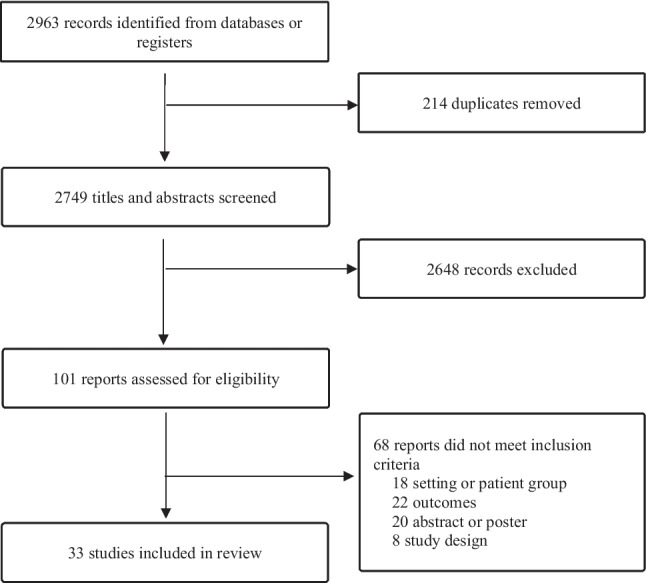
Study selection

Of 2963 unique articles screened, 33 met the inclusion criteria (Fig. 1). Table 1 summarises key study characteristics. Most reports came from the UK (14/33 [42%]), Denmark (6/33 [18%]), and the USA (4/33 [12%]). Twenty-one (64%) used national population-level data. The most common designs were cohort (14/33 [42%]) and observational (9/33 [27%]). Twenty-five studies included children, four focused on adolescents/young adults and four were literature reviews. Further study-level details are provided in Online Resource 4.

**Table 1:** Descriptive characteristics of 33 articles included in the review

	Number (%)
Publication year	
2012–2015	9 (27%)
2016–2019	8 (24%)
2020–2025	16 (48%)
Setting	
UK	14 (42%)
Denmark	6 (18%)
USA	4 (12%)
Multi-country	3 (9%)
Brazil	2 (6%)
Germany	1 (3%)
Italy	1 (3%)
Spain	1 (3%)
Sweden	1 (3%)
Population studied	
National population-level data	21 (64%)
Regional-level data	5 (15%)
Not stated	4 (12%)
Patient-level data	3 (9%)
Study type	
Observational	28 (85%)
Cohort	14 (42%)
Case–control	4 (12%)
Cross sectional	2 (6%)
Not specified	8 (24%)
Review	3 (9%)
Systematic review and meta-analysis	1 (3%)
Exploratory	1 (3%)
Age range of the population studied*	
Children and teenagers	10 (30%)
Children, teenagers, and young adults	9 (27%)
Children, teenagers, young adults, and adults	4 (12%)
Teenagers and young adults	4 (12%)
Not applicable (literature reviews or reviews)	4 (12%)
Children and adults	2 (6%)

*Age definitions used are described in Table 2

**Table 2:** Time intervals and number of visits before diagnosis identified in the scoping review

Age range (yrs)	Time intervals in children, teenagers, and young adults	Ref
0–18	CNS tumours only, Swedish national cohort diagnosed 2013–2016 Median total interval (symptom onset to start of treatment): 9.9 weeks (IQR 3.7–30.2) Median total diagnostic interval (symptom onset to diagnosis): 8.3 weeks (IQR 2.7–25.3) Median patient interval (symptom onset to presentation at a health-care facility): 2.6 weeks (IQR 0.3–8.4) Median primary care interval (presentation at a health-care facility to referral to an oncology centre): 1.7 weeks (IQR 0.1–12.0)	[39]
Not stated	CNS tumours only, data collected from 18 children’s cancer centres in UK Following a UK national awareness campaign called “HeadSmart: Be Brain Tumour Aware”, the median total diagnostic interval reduced from 9.1 weeks in 2011 to 6.7 weeks in the second year post-launch (2012–2013) The median diagnostic interval (from first medical contact to CNS imaging) reduced from 3.3 weeks to 1.4 weeks (*p* = 0.009) No significant change in patient interval (first symptom to first presentation)	[35]
Children	All cancers, retrospective audit of paediatricians in a single region in Italy Median diagnostic interval (symptom onset to diagnosis): 14 days (range: a few hours to 1095 days). Median for leukaemias = 7 days and for solid tumours, 20 days Estimated number of new children with cancer per paediatrician was 0.17 per year	[29]
0–25	CNS tumours only, USA single institution, diagnosed 2008–2017 Median total diagnostic interval (TDI): 42 days (IQR 14–120) Median TDI longer 150 days (IQR 60–135) in patients without a recognised cancer predisposition syndrome Evidence of significant differences in total diagnostic interval by tumour location and age	[32]
0–24	Multiple cancer types, England GP research database (1300 practices), diagnosed 1998–2018 Median total diagnostic interval (from first medical contact to diagnosis) for any symptom: 41 days Childhood cases (0–14 years old) had 25 associated symptoms with a median diagnostic time interval of 38 days In teenagers and young adults (15–24 years old), 20 symptoms were identified, and the median diagnostic interval was 44 days	[38]
0–18	Prospective study of all new cancer cases (*n* = 1933) presenting to the national network of children’s cancer treatment centres in the UK, 2020–2023 Median total diagnostic interval: 4.6 weeks (IQR 2.0–11.4) Median symptom interval (parent-reported to first medical contact): 1.1 weeks (IQR 0.1–4.0) Median system interval (first presentation to diagnosis): 1.7 weeks (IQR 0.4–5.9) Median TDI longest in 15–18 years (8.7 weeks, IQR 3.0–17.4) and in bone tumours (12.6 weeks, IQR 6.6–23.4)	[41]
	**Number of visits before cancer diagnosis**	
4–18	Bone tumours (Ewing or Osteosarcoma), USA, Institutional retrospective audit. diagnosed 2004–2020 Mean number of encounters with the health-care system before sarcoma diagnosis: 1.9 ± 0.6 (range 1–4)	[11]
15–24	All cancers, England (national GP research database, 600 practices) diagnosis 1988–2010 In the 12 months before diagnosis, cases had a median of 5 (IQR 3–9) consultations compared with 2 (IQR 0–4) in controls Differences in consultation rates were most apparent in the 3 months before diagnosis cases having a median of 3 consultations (IQR 1–5) compared with 0 consultations in controls (IQR 0–1)	[18]
0–15	All cancers, Denmark (national), diagnosis 2008–2015 75.3% of children with cancer consulted the general practice within 3 months before diagnosis Socioeconomic gradient in frequency of consultations with highest odds of frequent use of consultations among children from low income (odds ratio 1.94 (95% CI 1.24–3.03) compared to high income families Consultations increased significantly from 16 to 18 months before diagnosis, with a progressive rise from 10 to 12 months before, especially in the last 3 months	[30]
0–25	Average number of health-care provider (HCP) visits before diagnosis of a brain tumour in patients that did not have a tumor predisposition syndrome was 2.4	[32]
0–14	All cancers, England (national GP research database, 600 practices) diagnosis 1988–2010 Median number of consultations in the 3 months before diagnosis: 3 (IQR 2–4) compared with 1 (IQR 1–2) in controls 35.5% had ≥ 4 consultations compared with 9.1% of controls	[19]
0–24	Brain tumours. UK. Diagnosis 1989–2006 Presentation rate to hospital increased from 1.3 per 100 person-months (95% CI 1.1 to 1.4) 6–12 months before diagnosis to 6.4 per 100 person-months (5.8 to 7.0) 1–3 months before, rising steeply to 134.0 (130.4 to 137.6) in the final month before diagnosis. Corresponding rates for presentation to primary care were 8.4 (95% CI 6.8–10.5), 26.7 (21.7–32.8), 148.9 (131.9–168.1) The proportion of emergency presentations to hospitals for children with brain tumours rose steadily from 35% over 12 months before diagnosis to 55% by the time of diagnosis Patients were seen in primary care over four times as often as in hospital up to the final month before the tumour was diagnosed	[26]
15–39 yrs	All cancers, Denmark (national), diagnosed 2002–2011 A progressive increase in consultations was observed for teenage and young adult cases, especially during the last 3 months before cancer diagnosis	[22]
0–24 yrs	Brain tumours, UK, diagnosed 1989–2006 Consultation rates in primary care peaked in the final month before diagnosis at rates over 100 consultations per 100 person months Hospital admissions were most frequent within 1 month before diagnosis with rates over 100 admissions per 100 person months	[27]
0–15 yrs	All cancers, Denmark, diagnosed 1998–2016 The number of contacts with health care was particularly high during the last 3 months before diagnosis, 47% of children showed frequent contacts (≥ 8), and 43% had frequent emergency contacts (≥ 2)	[36]
0–18 yrs	All cancers, UK, presentation 2020–2023 Majority (74%) had 1–3 pre-referral consultations Primary care most common first point of contact (59.4%); 20.7% presented via emergency care, only 5% first presented to a paediatrician	[41]
0–39 yrs	Sarcomas, Denmark (National), diagnosed 1997–2020 Sarcoma patients more likely than the background Danish population to have contacts in the primary healthcare sector, hospital outpatient clinics, and emergency rooms, and to fill prescriptions for pain- and antimicrobial medication for up to 24 consecutive months before diagnosis	[40]

Measures of variability (median and range, standard deviation (SD), interquartile range (IQR), or 95% CI) are reported as provided in the source publication; where a source publication did not report a variability measure, none is shown. Additional information is available in the appendix (Online resource 4) and the cited reference

*CNS* central nervous system

Three central themes emerged: (1) the healthcare-seeking behaviours of families and their interface with the diagnostic system; (2) awareness and recognition of alarm symptoms by parents, healthcare professionals, or both; and (3) system/training factors influencing referral and diagnosis.

### Healthcare-seeking behaviours of families and their interface with the diagnostic system

Twenty-six studies examined how families accessed care and how children and adolescents entered the diagnostic pathway [10, 11, 18–41]. Children were most often brought for assessment following parental concerns, sometimes for serious signs (e.g. lumps and bruising), but more often for vague symptoms (fatigue, fever, and pain). Families accessed care through a range of routes: community-based paediatricians, general practitioners (GPs), or urgent and out-of-hours hospital visits, frequently initiated directly by the parent. In several studies, emergency department (ED) visits occurred after multiple prior contacts [10, 23, 31, 33]. Cancers were less frequently detected during routine child surveillance or screening in at-risk populations. In a Danish registry study, children and young people with sarcomas were more likely than the background population to have contacts in the primary healthcare sector, hospital outpatients, and ED, and to fill prescriptions for pain- and antimicrobial medication for up to 24 consecutive months before diagnosis [40]. In a recent prospective national UK study, only 5% of children with cancer were diagnosed through interaction with a paediatrician [41].

The family-healthcare interface plays a central role in diagnostic timing. There was considerable variation in where and how children first presented, as well as how symptoms were interpreted. Although GPs may only encounter 1–2 childhood cancer cases in a career [20, 29, 42], they were often involved as first assessors or referrers [10, 18, 21, 23, 25, 30, 34, 39].

Diagnostic intervals varied widely, influenced by referral systems, symptom interpretation, tumour type, emergency presentation, and healthcare access. Table 2 summarises these data.

### Awareness and recognition of alarm symptoms

Fifteen studies reported on symptom recognition [18–21, 24–28, 32, 34, 35, 37–39]. Non-specific symptoms were common, mimicking benign illness, particularly in primary care, and delaying diagnosis. Several studies noted increased primary care consultations prior to diagnosis [19, 26, 27, 39].

UK and Danish studies identified early symptoms (pain, pallor, and fatigue) and high PPV signs (e.g. seizures, persistent lumps) [18–21, 26]. Two UK linkage studies reported seizures and abnormal movements had the highest PPVs for CNS tumours, and lump, mass, or swelling below the neck, excluding the abdomen, had the highest PPV for bone or tissue sarcomas [18, 20]. One study emphasised that individual symptoms and consultation patterns had low PPV in primary care [38].

Clinician awareness was often limited. Although most Italian primary care paediatricians reported involvement in the diagnostic process, few felt confident in identifying cancer early [29]. In Brazil, lack of formal paediatric training correlated with reduced suspicion of cancer [34].

Studies of CNS tumours described weeks or months of persistent symptoms—headache, vomiting, and behaviour change—before referral [39]. UK registry-linkage studies showed increased primary care visits for neurological symptoms in the year before diagnosis [26, 27].

The UK HeadSmart campaign, combining guidelines and a symptom checklist, was linked to diagnostic interval reductions from a median of 14 to 6.7 weeks [35]. While positively received by paediatricians, GPs were less engaged [24]. Spanish-language reviews and the PAHO/WHO “Early Diagnosis” manual also emphasised the value of structured tools for recognising alarm symptoms [37, 43].

### Health system and training-related factors

This was described in 30 studies and included limited awareness and incorrect interpretation of presenting symptoms by both community-based healthcare professionals and parents, healthcare professionals’ lack of knowledge of paediatric referral pathways, and lack of formal training in paediatrics [10, 11, 18–20, 22–26, 29–41, 44–50]. The non-specificity of symptoms and lack of understanding of the features of cancer in children, teenagers, and young adults may have caused a delay in diagnosis. Non-specific symptoms alone were often insufficient triggers for referral [10, 11, 20, 23, 31, 33]. Sociodemographic factors such as older age, male sex, economic disadvantage, and lower educational level contributed to a longer time to diagnosis [30, 33, 36]. Moreover, lack of knowledge of diagnostic pathways, involvement of multiple health practitioners, and lack of public confidence in health systems impacted the time to diagnosis.

In Italy, > 80% of paediatrician-assessed children were promptly referred to hospital [29]. In contrast, German rural areas with limited paediatricians relied on GPs/internal medicine doctors, and training differences impacted diagnostic timelines [45]. In the UK, avoidable delays were attributed to primary care (49%) and secondary care (38%) phases of the pathway [10, 46].

### Clinician survey

The questions focused on gathering information on national parameters covering mandatory paediatric training of front-line healthcare practitioners, the presence of professional organisations and routine child health surveillance programmes, and information regarding the number of health checks and provision of information regarding alarm signs/symptoms. In total, 54 clinicians (one GP and one paediatrician from each of 27 countries) were invited to participate. Of these, 49 (91%) completed the questionnaire (Online resource 5), representing 23 countries; no responses were received from Austria, Belgium, France, or the Netherlands, for which publicly available data sources were used.

Table 3 summarises two key aspects of healthcare systems for young children: the frequency of routine child health checks that include a full physical examination conducted by a medically qualified doctor (not nurse) for children under 5 yrs and the usual healthcare practitioner providing initial assessment of a symptomatic young child. The number of routine child health checks ranged from 2 to 21 (median 10). These were conducted by either GPs or office paediatricians.

**Table 3:** Intensity of child health surveillance and usual first-contact health care practitioner by country (BENCHISTA Project participants)

	Number of full physical examinations within routine child health practices freely provided for children < 5 years	Intensity of child health surveillance*	Usual healthcare practitioner providing initial assessment of symptomatic young child
Australia	2	Low	GP
Ireland	2	Low	GP
UK	2	Low	GP
Norway	4	Low	GP
Sweden	4	Low	GP
Japan	5	Medium	Paediatrician
Malta	5	Medium	GP
Denmark	7	Medium	GP
Slovenia	8	Medium	Paediatrician
Hungary	9	Medium	Paediatrician
Switzerland	9	Medium	Paediatrician
Austria	10	High	Paediatrician
Estonia	10	High	GP
Germany	10	High	Paediatrician
Greece	10	High	Paediatrician
Poland	10	High	Paediatrician
Spain	10	High	Paediatrician
Canada	11	High	GP or community paediatrician or nurse; varies in urban vs rural settings
Romania	11	High	GP
Belgium	12	High	Health-care nurse or paediatrician
Brazil	12	High	GP
Czech Republic	12	High	Paediatrician
France	14	High	Paediatrician
Portugal	14	High	GP
Netherlands	14	High	Youth health-care nurses followed by youth doctors
Italy	15	High	Paediatrician
Bulgaria	21	High	GP

*Classification of countries as low (< 5), medium (5–9), or high (≥ 10) is based on the number of universally offered child health checks that include a full physical examination conducted by a doctor (not nurse) and was assigned by the study authors

*GP* general practitioner

Similar numbers of countries reported either GPs (*n* = 12) or paediatricians (*n* = 12) as the first usual point of contact for acute illness, with three countries reporting a mixed picture according to geography or integration with community nurses (Table 3). In the survey, mandatory post-graduate paediatric training as part of qualification as a first-contact clinician (GP/family doctor) was consistently reported in 15 of 23 countries (65%), with discrepant answers in 3 (13%). Three countries did not include mandatory paediatric training (Japan, Norway, UK). Only 4/23 countries reported provision of written information to new parents on alarm signs and symptoms of serious childhood illnesses, which could include cancer, whereas most child health booklets included expected developmental milestones (Online resource 6). Respondents noted gaps in paediatric training programmes, continuity of primary and emergency care, and coordination between primary and secondary services. These themes were also noted in the papers included in the literature review and are summarised in online resource 4.

## Discussion

We identified three central components influencing the timeliness of childhood cancer diagnosis across BENCHISTA countries: (1) healthcare-seeking behaviour of families and the diagnostic interface with healthcare professionals, particularly in primary care; (2) awareness and interpretation of alarm symptoms by both caregivers and healthcare providers; and (3) system-level and training-related factors. Although national practices varied, shared barriers within these domains may contribute to delayed recognition, referral, and treatment.

Multiple studies reported a pattern of increased primary care consultations before cancer diagnosis, often for vague symptoms [19, 26, 27, 39, 41]. Children often presented with vague or non-specific symptoms, which led to misdiagnoses, repeated visits, and prolonged time to diagnosis. This suggests that primary care professionals, particularly general practitioners, need better support to recognise and act on early warning signs.

Low awareness of paediatric cancer symptoms and insufficient training were recurrent themes. This knowledge gap is especially pronounced in settings with limited paediatric training. In one study from an LMIC, fewer than half of GPs in Brazil reported receiving formal paediatric training, contributing to low rates of routine child examination and reduced diagnostic confidence [34]. These findings highlight opportunities for targeted education, structured training, and decision-support tools for frontline providers [51–53].

In response to diagnostic delay, the UK’s HeadSmart campaign implemented an awareness strategy with referral tools, reducing diagnostic intervals for CNS tumours [35]. The recent Childhood Cancer Diagnosis Study confirmed that children often had multiple consultations before diagnosis, particularly for non-specific symptoms [41]. While public and professional awareness campaigns improved diagnostic intervals in some tumour groups, missed opportunities remained. The study concluded that symptom recognition alone is insufficient, and highlighted the need for decision-support tools, training, and more structured pathways in primary care [41]. These insights align with our international review, highlighting the importance of system-level investment, clinician preparedness, and family–clinician communication in achieving earlier diagnosis. Delayed diagnosis, especially emergency presentations, has been linked to poorer outcomes. Broader challenges include fragmented health systems, and socio-cultural factors affecting clinician–caregiver interactions. Such system-level challenges highlight the importance of strengthening primary care infrastructure for children, improving care coordination, and addressing social determinants of health.

Survey findings revealed wide variation between countries in child health surveillance, the requirement for paediatric training for healthcare professionals, and national guidelines addressing alarm symptoms (Table 3 and Online resources 5 & 6). Although most countries rely on GPs as the first point of contact for serious childhood illness, surveillance intensity and referral frameworks vary substantially. For example, Bulgaria reported a high number of routine child health checks, but the quality of care and diagnostic pathways remain variable, mostly due to geographical and financial barriers, despite recent reforms [54].

In our survey, 13 of 23 European countries reported 10 or more routine physical examinations by a medically qualified practitioner for children under 5 years of age. This suggests a strong surveillance infrastructure, yet countries with lower survival rates in the EUROCARE study particularly in Eastern Europe [7] report some of the highest examination frequencies (Table 3). This disconnect implies that frequent surveillance alone may not ensure timely diagnosis. Other barriers, such as delayed access to hospital-based investigations or slow referral to specialist centres may play a role. A BENCHISTA analysis of geographic stage distribution also noted variation in use of staging investigations [15], likely reflecting limitations in diagnostic infrastructure.

Most BENCHISTA countries are HICs, yet our findings mirrored challenges reported in LMICs. The PAHO/WHO “Early Diagnosis” manual describes similar issues—low awareness, lack of structured referral, and training gaps [43]. Similar barriers to timely diagnosis and treatment of childhood cancer in Sub-Sahara Africa were recently described [55]. This underscores the need for shared global approaches to early diagnosis.

Our study has several strengths. Our study integrates a scoping review and cross-country survey, providing a comprehensive view of how child health systems shape diagnostic pathways. However, as a scoping review and cross-sectional survey, our findings should be interpreted as descriptive rather than causal. Limitations include exclusion of non-English/Spanish studies and overrepresentation of HICs. Questionnaire responses may reflect language or reporting bias. We addressed this by triangulating with national guidance and literature.

Our findings suggest opportunities for future research. Combining our descriptive findings with BENCHISTA registry data on stage and survival may help explain international differences in childhood cancer outcomes [15, 16, 47]. Further work should explore standardised indicators for primary care quality, electronic health-record integration across primary and secondary care, qualitative studies of diagnostic delay, and the potential impact of structured diagnostic decision-support tools and investment in training—particularly in systems where first-contact clinicians do not complete mandatory paediatric training. These findings complement earlier work, including a 2013 European review by Wolfe et al., which reported wide variability in child health systems and slower progress in child mortality reduction in the UK than in comparable countries, suggesting that the structure of paediatric care matters [13]. Harmonised and timely diagnostic pathways may contribute to improved equity in early cancer diagnosis and less variable survival outcomes for children across BENCHISTA countries and beyond.

## Supplementary Information

Below is the link to the electronic supplementary material.

ESM 1 (PDF 903 KB)

## Data Availability

All data supporting the findings of this study are available within the paper and its Supplementary Information.

## Abbreviations

BENCHISTA: International Benchmarking of Childhood Cancer Survival by Stage
CNS: Central nervous system
CI: Confidence interval
GP: General practitioner
HIC: High-income country
IQR: Interquartile range
LMIC: Low- and middle-income countries
NHS: National Health Service
PPV: Positive predictive value
PWG: BENCHISTA project working group
UCL: University College London

## Declarations

### Patient, parent, and public involvement and engagement statement

The BENCHISTA project involves parents and guardians of children with cancer, who are represented in the project working group and the independent advisory board.
