# Correction to: International variation in child healthcare practices and implications for childhood cancer diagnosis: a scoping review and cross-country clinician survey

**DOI:** 10.1007/s00431-026-07238-1

**Published:** 2026-07-20

**Authors:** Angela Lopez-Cortes, Aisha Al-Mahdy, Zsuzsanna Jakab, Masako Shimato, Laura Botta, Fabio Didonè, Adela Cañete Nieto, Emmanuel Desandes, Lisa L. Hjalgrim, Angela Polanco, Charles A. Stiller, Bernward Zeller, Gemma Gatta, Kathy Pritchard-Jones, The BENCHISTA Project Working Group

**Affiliations:** 1 UCL Developmental Biology & Cancer Research Department, UCL GOS Institute of Child Health, London, UK; 2 National Childhood Cancer Registry (NCCR), Hungarian Pediatric Oncology Network (HuPON), Budapest, Hungary; 3 Department of Pediatrics, Semmelweis University, Budapest, Hungary; 4 Evaluative Epidemiology Unit, Department of Epidemiology and Data Science, Fondazione IRCCS Istituto Nazionale Dei Tumori, Milan, Italy; 5 Paediatric Oncology and Hematology Unit, Dept. of Pediatrics, Obstetrics and Gynecoloy, Hospital UiP La Fe, Valencia, Spain; 6 Spanish Registry of Childhood Tumours (RETI-SEHOP), University of Valencia, Valencia, Spain; 7 National Registry of Childhood Cancers, CRESS, UMRS 1153, INSERM, Paris-Cité University, Paris, France; 8 National Registry of Childhood Solid Tumors, Institut de Cancérologie de Lorraine, Vandœuvre-Lès-Nancy, France; 9 Department of Paediatrics and Adolescent Medicine, University of Copenhagen, Rigshospitalet, Copenhagen, Denmark; 10 CCLG: The Children & Young People’s Cancer Association, Leicester, UK; 11 National Disease Registration Service, Transformation Directorate, NHS England, London, UK; 12 Division of Paediatrics and Adolescent Medicine, Oslo University Hospital, Oslo, Norway; 13 Department of Statistics and Quantitative Methods, University of Milano-Bicocca, Milan, Italy

**Correction to: European Journal of Pediatrics (2026) 185:491**

10.1007/s00431-026-07109-9

An erratum has been published for the article titled "International variation in child healthcare practices and implications for childhood cancer diagnosis: a scoping review and cross-country clinician survey".

**Table 1**

- **Published form:** Four lines of study types with their associated percentage of all the Observation studies are displayed as part of a continuous list.

**Table 1:** Descriptive characteristics of 33 articles included in the review

	Number (%)
Publication year	
2012–2015	9 (27%)
2016–2019	8 (24%)
2020–2025	16 (48%)
Setting	
UK	14 (42%)
Denmark	6 (18%)
USA	4 (12%)
Multi-country	3 (9%)
Brazil	2 (6%)
Germany	1 (3%)
Italy	1 (3%)
Spain	1 (3%)
Sweden	1 (3%)
Population studied	
National population-level data	21 (64%)
Regional-level data	5 (15%)
Not stated	4 (12%)
Patient-level data	3 (9%)
Study type	
Observational	28 (85%)
Cohort	14 (42%)
Case–control	4 (12%)
Cross sectional	2 (6%)
Not specified	8 (24%)
Review	3 (9%)
Systematic review and meta-analysis	1 (3%)
Exploratory	1 (3%)
Age range of the population studied*	
Children and teenagers	10 (30%)
Children, teenagers, and young adults	9 (27%)
Children, teenagers, young adults, and adults	4 (12%)
Teenagers and young adults	4 (12%)
Not applicable (literature reviews or reviews)	4 (12%)
Children and adults	2 (6%)

*Age definitions used are described in Table 2

- **Corrected form:** These four lines of study types and their associated percentages have been indented to properly show they are sub-categories under Observation studies, correcting the total percentage calculation.

**Table 1:** Descriptive characteristics of 33 articles included in the review

	Number (%)
Publication year	
2012–2015	9 (27%)
2016–2019	8 (24%)
2020–2025	16 (48%)
Setting	
UK	14 (42%)
Denmark	6 (18%)
USA	4 (12%)
Multi-country	3 (9%)
Brazil	2 (6%)
Germany	1 (3%)
Italy	1 (3%)
Spain	1 (3%)
Sweden	1 (3%)
Population studied	
National population-level data	21 (64%)
Regional-level data	5 (15%)
Not stated	4 (12%)
Patient-level data	3 (9%)
Study type	
Observational	28 (85%)
Cohort	14 (42%)
Case–control	4 (12%)
Cross sectional	2 (6%)
Not specified	8 (24%)
Review	3 (9%)
Systematic review and meta-analysis	1 (3%)
Exploratory	1 (3%)
Age range of the population studied*	
Children and teenagers	10 (30%)
Children, teenagers, and young adults	9 (27%)
Children, teenagers, young adults, and adults	4 (12%)
Teenagers and young adults	4 (12%)
Not applicable (literature reviews or reviews)	4 (12%)
Children and adults	2 (6%)

*Age definitions used are described in Table 2

**Table 2**

- **Published form:** "Median primary care interval (presentation at a health-care facility to referral to an oncology centre): 1 × 7 weeks (IQR 0.1–12.0)"

**Table 2:** Time intervals and number of visits before diagnosis identified in the scoping review

Age range (yrs)	Time intervals in children, teenagers, and young adults	Ref.
0-18	CNS tumours only, Swedish national cohort diagnosed 2013–2016 Median total interval (symptom onset to start of treatment): 9.9 weeks (IQR 3.7–30.2) Median total diagnostic interval (symptom onset to diagnosis): 8.3 weeks (IQR 2.7–25.3) Median patient interval (symptom onset to presentation at a health-care facility): 2.6 weeks (IQR 0.3–8.4) Median primary care interval (presentation at a health-care facility to referral to an oncology centre): 1 × 7 weeks (IQR 0.1–12.0)	[39]
Not stated	CNS tumours only, data collected from 18 children’s cancer centres in UK Following a UK national awareness campaign called “HeadSmart: Be Brain Tumour Aware”, the median total diagnostic interval reduced from 9.1 weeks in 2011 to 6.7 weeks in the second year post-launch (2012–2013) The median diagnostic interval (from first medical contact to CNS imaging) reduced from 3.3 weeks to 1.4 weeks (p = 0.009) No significant change in patient interval (first symptom to first presentation)	[35]
Children	All cancers, retrospective audit of paediatricians in a single region in Italy All cancers, retrospective audit of paediatricians in a single region in Italy Estimated number of new children with cancer per paediatrician was 0.17 per year	[29]
0–25	CNS tumours only, USA single institution, diagnosed 2008–2017 Median total diagnostic interval (TDI): 42 days (IQR 14–120) Median TDI longer 150 days (IQR 60–135) in patients without a recognised cancer predisposition syndrome Evidence of significant differences in total diagnostic interval by tumour location and age	[32]
0–24	Multiple cancer types, England GP research database (1300 practices), diagnosed 1998–2018 Median total diagnostic interval (from first medical contact to diagnosis) for any symptom: 41 days Childhood cases (0–14 years old) had 25 associated symptoms with a median diagnostic time interval of 38 days In teenagers and young adults (15–24 years old), 20 symptoms were identified, and the median diagnostic interval was 44 days	[38]
0–18	Prospective study of all new cancer cases (n = 1933) presenting to the national network of children’s cancer treatment centres in the UK, 2020–2023 Median total diagnostic interval: 4.6 weeks (IQR 2.0–11.4) Median symptom interval (parent-reported to first medical contact): 1.1 weeks (IQR 0.1–4.0) Median system interval (first presentation to diagnosis): 1.7 weeks (IQR 0.4–5.9) Median TDI longest in 15–18 years (8.7 weeks, IQR 3.0–17.4) and in bone tumours (12.6 weeks, IQR 6.6–23.4)	[41]
	Number of visits before cancer diagnosis	
4–18	Bone tumours (Ewing or Osteosarcoma), USA, Institutional retrospective audit. diagnosed 2004–2020 Mean number of encounters with the health-care system before sarcoma diagnosis: 1.9 ± 0.6 (range 1–4)	[11]
15–24	All cancers, England (national GP research database, 600 practices) diagnosis 1988–2010 In the 12 months before diagnosis, cases had a median of 5 (IQR 3–9) consultations compared with 2 (IQR 0–4) in controls Differences in consultation rates were most apparent in the 3 months before diagnosis cases having a median of 3 consultations (IQR 1–5) compared with 0 consultations in controls (IQR 0–1)	[18]
0–15	All cancers, Denmark (national), diagnosis 2008–2015 75,3% of children with cancer consulted the general practice within 3 months before diagnosis Socioeconomic gradient in frequency of consultations with highest odds of frequent use of consultations among children from low income (odds ratio 1.94 (95% CI 1.24–3.03) compared to high income families Consultations increased significantly from 16 to 18 months before diagnosis, with a progressive rise from 10 to 12 months before, especially in the last 3 months	[30]
0–25	Average number of health-care provider (HCP) visits before diagnosis of a brain tumour in patients that did not have a tumor predisposition syndrome was 2.4	[32]
0–14	All cancers, England (national GP research database, 600 practices) diagnosis 1988–2010 Median number of consultations in the 3 months before diagnosis: 3 (IQR 2–4) compared with 1 (IQR 1–2) in controls 35.5% had ≥ 4 consultations compared with 9.1% of controls	[19]
0–24	Brain tumours. UK. Diagnosis 1989–2006 Presentation rate to hospital increased from 1.3 per 100 person-months (95% CI 1.1 to 1.4) 6–12 months before diagnosis to 6.4 per 100 person-months (5.8 to 7.0) 1–3 months before, rising steeply to 134.0 (130.4 to 137.6) in the final month before diagnosis. Corresponding rates for presentation to primary care were 8.4 (95% CI 6.8–10.5), 26.7 (21.7–32.8), 148.9 (131.9–168.1) The proportion of emergency presentations to hospitals for children with brain tumours rose steadily from 35% over 12 months before diagnosis to 55% by the time of diagnosis Patients were seen in primary care over four times as often as in hospital up to the final month before the tumour was diagnosed	[26]
15–39 yrs	All cancers, Denmark (national), diagnosed 2002–2011 A progressive increase in consultations was observed for teenage and young adult cases, especially during the last 3 months before cancer diagnosis	[22]
0–24 yrs	Brain tumours, UK, diagnosed 1989–2006 Consultation rates in primary care peaked in the final month before diagnosis at rates over 100 consultations per 100 person months Hospital admissions were most frequent within 1 month before diagnosis with rates over 100 admissions per 100 person months	[27]
0–15 yrs	All cancers, Denmark, diagnosed 1998–2016 The number of contacts with health care was particularly high during the last 3 months before diagnosis, 47% of children showed frequent contacts (≥ 8), and 43% had frequent emergency contacts (≥ 2)	[36]
0–18 yrs	All cancers, UK, presentation 2020–2023 Majority (74%) had 1–3 pre-referral consultations Majority (74%) had 1–3 pre-referral consultations	[41]
0–39 yrs	Sarcomas, Denmark (National), diagnosed 1997–2020 Sarcoma patients more likely than the background Danish population to have contacts in the primary healthcare sector, hospital outpatient clinics, and emergency rooms, and to fill prescriptions for pain- and antimicrobial medication for up to 24 consecutive months before diagnosis	[40]

Measures of variability (median and range, standard deviation (SD), interquartile range (IQR), or 95% CI) are reported as provided in the source publication; where a source publication did not report a variability measure, none is shown. Additional information is available in the appendix (Online resource 4) and the cited reference

*CNS* central nervous system

- **Corrected form:**
  "Median primary care interval (presentation at a health-care facility to referral to an oncology centre): 1.7 weeks (IQR 0.1–12.0)"

**Table 2:** Time intervals and number of visits before diagnosis identified in the scoping review

Age range (yrs)	Time intervals in children, teenagers, and young adults	Ref.
0-18	CNS tumours only, Swedish national cohort diagnosed 2013–2016 Median total interval (symptom onset to start of treatment): 9.9 weeks (IQR 3.7–30.2) Median total diagnostic interval (symptom onset to diagnosis): 8.3 weeks (IQR 2.7–25.3) Median patient interval (symptom onset to presentation at a health-care facility): 2.6 weeks (IQR 0.3–8.4) Median primary care interval (presentation at a health-care facility to referral to an oncology centre): 1.7 weeks (IQR 0.1–12.0)	[39]
Not stated	CNS tumours only, data collected from 18 children’s cancer centres in UK Following a UK national awareness campaign called “HeadSmart: Be Brain Tumour Aware”, the median total diagnostic interval reduced from 9.1 weeks in 2011 to 6.7 weeks in the second year post-launch (2012–2013) The median diagnostic interval (from first medical contact to CNS imaging) reduced from 3.3 weeks to 1.4 weeks (p = 0.009) No significant change in patient interval (first symptom to first presentation)	[35]
Children	All cancers, retrospective audit of paediatricians in a single region in Italy Median diagnostic interval (symptom onset to diagnosis): 14 days (range: a few hours to 1095 days). Median for leukaemias = 7 days and for solid tumours, 20 days Estimated number of new children with cancer per paediatrician was 0.17 per year	[29]
0–25	CNS tumours only, USA single institution, diagnosed 2008–2017 Median total diagnostic interval (TDI): 42 days (IQR 14–120) Median TDI longer 150 days (IQR 60–135) in patients without a recognised cancer predisposition syndrome Evidence of significant differences in total diagnostic interval by tumour location and age	[32]
0–24	Multiple cancer types, England GP research database (1300 practices), diagnosed 1998–2018 Median total diagnostic interval (from first medical contact to diagnosis) for any symptom: 41 days Childhood cases (0–14 years old) had 25 associated symptoms with a median diagnostic time interval of 38 days In teenagers and young adults (15–24 years old), 20 symptoms were identified, and the median diagnostic interval was 44 days	[38]
0–18	Prospective study of all new cancer cases (n = 1933) presenting to the national network of children’s cancer treatment centres in the UK, 2020–2023 Median total diagnostic interval: 4.6 weeks (IQR 2.0–11.4) Median symptom interval (parent-reported to first medical contact): 1.1 weeks (IQR 0.1–4.0) Median system interval (first presentation to diagnosis): 1.7 weeks (IQR 0.4–5.9) Median TDI longest in 15–18 years (8.7 weeks, IQR 3.0–17.4) and in bone tumours (12.6 weeks, IQR 6.6–23.4)	[41]
	Number of visits before cancer diagnosis	
4–18	Bone tumours (Ewing or Osteosarcoma), USA, Institutional retrospective audit. diagnosed 2004–2020 Mean number of encounters with the health-care system before sarcoma diagnosis: 1.9 ± 0.6 (range 1–4)	[11]
15–24	All cancers, England (national GP research database, 600 practices) diagnosis 1988–2010 In the 12 months before diagnosis, cases had a median of 5 (IQR 3–9) consultations compared with 2 (IQR 0–4) in controls Differences in consultation rates were most apparent in the 3 months before diagnosis cases having a median of 3 consultations (IQR 1–5) compared with 0 consultations in controls (IQR 0–1)	[18]
0–15	All cancers, Denmark (national), diagnosis 2008–2015 75,3% of children with cancer consulted the general practice within 3 months before diagnosis Socioeconomic gradient in frequency of consultations with highest odds of frequent use of consultations among children from low income (odds ratio 1.94 (95% CI 1.24–3.03) compared to high income families Consultations increased significantly from 16 to 18 months before diagnosis, with a progressive rise from 10 to 12 months before, especially in the last 3 months	[30]
0–25	Average number of health-care provider (HCP) visits before diagnosis of a brain tumour in patients that did not have a tumor predisposition syndrome was 2.4	[32]
0–14	All cancers, England (national GP research database, 600 practices) diagnosis 1988–2010 Median number of consultations in the 3 months before diagnosis: 3 (IQR 2–4) compared with 1 (IQR 1–2) in controls 35.5% had ≥ 4 consultations compared with 9.1% of controls	[19]
0–24	Brain tumours. UK. Diagnosis 1989–2006 Presentation rate to hospital increased from 1.3 per 100 person-months (95% CI 1.1 to 1.4) 6–12 months before diagnosis to 6.4 per 100 person-months (5.8 to 7.0) 1–3 months before, rising steeply to 134.0 (130.4 to 137.6) in the final month before diagnosis. Corresponding rates for presentation to primary care were 8.4 (95% CI 6.8–10.5), 26.7 (21.7–32.8), 148.9 (131.9–168.1) The proportion of emergency presentations to hospitals for children with brain tumours rose steadily from 35% over 12 months before diagnosis to 55% by the time of diagnosis Patients were seen in primary care over four times as often as in hospital up to the final month before the tumour was diagnosed	[26]
15–39 yrs	All cancers, Denmark (national), diagnosed 2002–2011 A progressive increase in consultations was observed for teenage and young adult cases, especially during the last 3 months before cancer diagnosis	[22]
0–24 yrs	Brain tumours, UK, diagnosed 1989–2006 Consultation rates in primary care peaked in the final month before diagnosis at rates over 100 consultations per 100 person months Hospital admissions were most frequent within 1 month before diagnosis with rates over 100 admissions per 100 person months	[27]
0–15 yrs	All cancers, Denmark, diagnosed 1998–2016 The number of contacts with health care was particularly high during the last 3 months before diagnosis, 47% of children showed frequent contacts (≥ 8), and 43% had frequent emergency contacts (≥ 2)	[36]
0–18 yrs	All cancers, UK, presentation 2020–2023 Majority (74%) had 1–3 pre-referral consultations Majority (74%) had 1–3 pre-referral consultations	[41]
0–39 yrs	Sarcomas, Denmark (National), diagnosed 1997–2020 Sarcoma patients more likely than the background Danish population to have contacts in the primary healthcare sector, hospital outpatient clinics, and emergency rooms, and to fill prescriptions for pain- and antimicrobial medication for up to 24 consecutive months before diagnosis	[40]

Measures of variability (median and range, standard deviation (SD), interquartile range (IQR), or 95% CI) are reported as provided in the source publication; where a source publication did not report a variability measure, none is shown. Additional information is available in the appendix (Online resource 4) and the cited reference

*CNS* central nervous system

- **Published form:**
  "All cancers, Denmark (national), diagnosis 2008–2015 75,3% of children with cancer consulted the general practice within 3 months before diagnosis"

**Table 2:** Time intervals and number of visits before diagnosis identified in the scoping review

Age range (yrs)	Time intervals in children, teenagers, and young adults	Ref.
0-18	CNS tumours only, Swedish national cohort diagnosed 2013–2016 Median total interval (symptom onset to start of treatment): 9.9 weeks (IQR 3.7–30.2) Median total diagnostic interval (symptom onset to diagnosis): 8.3 weeks (IQR 2.7–25.3) Median patient interval (symptom onset to presentation at a health-care facility): 2.6 weeks (IQR 0.3–8.4) Median primary care interval (presentation at a health-care facility to referral to an oncology centre): 1 × 7 weeks (IQR 0.1–12.0)	[39]
Not stated	CNS tumours only, data collected from 18 children’s cancer centres in UK Following a UK national awareness campaign called “HeadSmart: Be Brain Tumour Aware”, the median total diagnostic interval reduced from 9.1 weeks in 2011 to 6.7 weeks in the second year post-launch (2012–2013) The median diagnostic interval (from first medical contact to CNS imaging) reduced from 3.3 weeks to 1.4 weeks (p = 0.009) No significant change in patient interval (first symptom to first presentation)	[35]
Children	All cancers, retrospective audit of paediatricians in a single region in Italy Median diagnostic interval (symptom onset to diagnosis): 14 days (range: a few hours to 1095 days). Median for leukaemias = 7 days and for solid tumours, 20 days Estimated number of new children with cancer per paediatrician was 0.17 per year	[29]
0–25	CNS tumours only, USA single institution, diagnosed 2008–2017 Median total diagnostic interval (TDI): 42 days (IQR 14–120) Median TDI longer 150 days (IQR 60–135) in patients without a recognised cancer predisposition syndrome Evidence of significant differences in total diagnostic interval by tumour location and age	[32]
0–24	Multiple cancer types, England GP research database (1300 practices), diagnosed 1998–2018 Median total diagnostic interval (from first medical contact to diagnosis) for any symptom: 41 days Childhood cases (0–14 years old) had 25 associated symptoms with a median diagnostic time interval of 38 days In teenagers and young adults (15–24 years old), 20 symptoms were identified, and the median diagnostic interval was 44 days	[38]
0–18	Prospective study of all new cancer cases (n = 1933) presenting to the national network of children’s cancer treatment centres in the UK, 2020–2023 Median total diagnostic interval: 4.6 weeks (IQR 2.0–11.4) Median symptom interval (parent-reported to first medical contact): 1.1 weeks (IQR 0.1–4.0) Median system interval (first presentation to diagnosis): 1.7 weeks (IQR 0.4–5.9) Median TDI longest in 15–18 years (8.7 weeks, IQR 3.0–17.4) and in bone tumours (12.6 weeks, IQR 6.6–23.4)	[41]
	Number of visits before cancer diagnosis	
4–18	Bone tumours (Ewing or Osteosarcoma), USA, Institutional retrospective audit. diagnosed 2004–2020 Mean number of encounters with the health-care system before sarcoma diagnosis: 1.9 ± 0.6 (range 1–4)	[11]
15–24	All cancers, England (national GP research database, 600 practices) diagnosis 1988–2010 In the 12 months before diagnosis, cases had a median of 5 (IQR 3–9) consultations compared with 2 (IQR 0–4) in controls Differences in consultation rates were most apparent in the 3 months before diagnosis cases having a median of 3 consultations (IQR 1–5) compared with 0 consultations in controls (IQR 0–1)	[18]
0–15	All cancers, Denmark (national), diagnosis 2008–2015 75,3% of children with cancer consulted the general practice within 3 months before diagnosis Socioeconomic gradient in frequency of consultations with highest odds of frequent use of consultations among children from low income (odds ratio 1.94 (95% CI 1.24–3.03) compared to high income families Consultations increased significantly from 16 to 18 months before diagnosis, with a progressive rise from 10 to 12 months before, especially in the last 3 months	[30]
0–25	Average number of health-care provider (HCP) visits before diagnosis of a brain tumour in patients that did not have a tumor predisposition syndrome was 2.4	[32]
0–14	All cancers, England (national GP research database, 600 practices) diagnosis 1988–2010 Median number of consultations in the 3 months before diagnosis: 3 (IQR 2–4) compared with 1 (IQR 1–2) in controls 35.5% had ≥ 4 consultations compared with 9.1% of controls	[19]
0–24	Brain tumours. UK. Diagnosis 1989–2006 Presentation rate to hospital increased from 1.3 per 100 person-months (95% CI 1.1 to 1.4) 6–12 months before diagnosis to 6.4 per 100 person-months (5.8 to 7.0) 1–3 months before, rising steeply to 134.0 (130.4 to 137.6) in the final month before diagnosis. Corresponding rates for presentation to primary care were 8.4 (95% CI 6.8–10.5), 26.7 (21.7–32.8), 148.9 (131.9–168.1) The proportion of emergency presentations to hospitals for children with brain tumours rose steadily from 35% over 12 months before diagnosis to 55% by the time of diagnosis Patients were seen in primary care over four times as often as in hospital up to the final month before the tumour was diagnosed	[26]
15–39 yrs	All cancers, Denmark (national), diagnosed 2002–2011 A progressive increase in consultations was observed for teenage and young adult cases, especially during the last 3 months before cancer diagnosis	[22]
0–24 yrs	Brain tumours, UK, diagnosed 1989–2006 Consultation rates in primary care peaked in the final month before diagnosis at rates over 100 consultations per 100 person months Hospital admissions were most frequent within 1 month before diagnosis with rates over 100 admissions per 100 person months	[27]
0–15 yrs	All cancers, Denmark, diagnosed 1998–2016 The number of contacts with health care was particularly high during the last 3 months before diagnosis, 47% of children showed frequent contacts (≥ 8), and 43% had frequent emergency contacts (≥ 2)	[36]
0–18 yrs	All cancers, UK, presentation 2020–2023 Majority (74%) had 1–3 pre-referral consultations Majority (74%) had 1–3 pre-referral consultations	[41]
0–39 yrs	Sarcomas, Denmark (National), diagnosed 1997–2020 Sarcoma patients more likely than the background Danish population to have contacts in the primary healthcare sector, hospital outpatient clinics, and emergency rooms, and to fill prescriptions for pain- and antimicrobial medication for up to 24 consecutive months before diagnosis	[40]

Measures of variability (median and range, standard deviation (SD), interquartile range (IQR), or 95% CI) are reported as provided in the source publication; where a source publication did not report a variability measure, none is shown. Additional information is available in the appendix (Online resource 4) and the cited reference

*CNS* central nervous system

- **Corrected form:**
  "All cancers, Denmark (national), diagnosis 2008–2015 75.3% of children with cancer consulted the general practice within 3 months before diagnosis"

**Table 2:** Time intervals and number of visits before diagnosis identified in the scoping review

Age range (yrs)	Time intervals in children, teenagers, and young adults	Ref.
0-18	CNS tumours only, Swedish national cohort diagnosed 2013–2016 Median total interval (symptom onset to start of treatment): 9.9 weeks (IQR 3.7–30.2) Median total diagnostic interval (symptom onset to diagnosis): 8.3 weeks (IQR 2.7–25.3) Median patient interval (symptom onset to presentation at a health-care facility): 2.6 weeks (IQR 0.3–8.4) Median primary care interval (presentation at a health-care facility to referral to an oncology centre): 1.7 weeks (IQR 0.1–12.0)	[39]
Not stated	CNS tumours only, data collected from 18 children’s cancer centres in UK Following a UK national awareness campaign called “HeadSmart: Be Brain Tumour Aware”, the median total diagnostic interval reduced from 9.1 weeks in 2011 to 6.7 weeks in the second year post-launch (2012–2013) The median diagnostic interval (from first medical contact to CNS imaging) reduced from 3.3 weeks to 1.4 weeks (p = 0.009) No significant change in patient interval (first symptom to first presentation)	[35]
Children	All cancers, retrospective audit of paediatricians in a single region in Italy Median diagnostic interval (symptom onset to diagnosis): 14 days (range: a few hours to 1095 days). Median for leukaemias = 7 days and for solid tumours, 20 days Estimated number of new children with cancer per paediatrician was 0.17 per year	[29]
0–25	CNS tumours only, USA single institution, diagnosed 2008–2017 Median total diagnostic interval (TDI): 42 days (IQR 14–120) Median TDI longer 150 days (IQR 60–135) in patients without a recognised cancer predisposition syndrome Evidence of significant differences in total diagnostic interval by tumour location and age	[32]
0–24	Multiple cancer types, England GP research database (1300 practices), diagnosed 1998–2018 Median total diagnostic interval (from first medical contact to diagnosis) for any symptom: 41 days Childhood cases (0–14 years old) had 25 associated symptoms with a median diagnostic time interval of 38 days In teenagers and young adults (15–24 years old), 20 symptoms were identified, and the median diagnostic interval was 44 days	[38]
0–18	Prospective study of all new cancer cases (n = 1933) presenting to the national network of children’s cancer treatment centres in the UK, 2020–2023 Median total diagnostic interval: 4.6 weeks (IQR 2.0–11.4) Median symptom interval (parent-reported to first medical contact): 1.1 weeks (IQR 0.1–4.0) Median system interval (first presentation to diagnosis): 1.7 weeks (IQR 0.4–5.9) Median TDI longest in 15–18 years (8.7 weeks, IQR 3.0–17.4) and in bone tumours (12.6 weeks, IQR 6.6–23.4)	[41]
	Number of visits before cancer diagnosis	
4–18	Bone tumours (Ewing or Osteosarcoma), USA, Institutional retrospective audit. diagnosed 2004–2020 Mean number of encounters with the health-care system before sarcoma diagnosis: 1.9 ± 0.6 (range 1–4)	[11]
15–24	All cancers, England (national GP research database, 600 practices) diagnosis 1988–2010 In the 12 months before diagnosis, cases had a median of 5 (IQR 3–9) consultations compared with 2 (IQR 0–4) in controls Differences in consultation rates were most apparent in the 3 months before diagnosis cases having a median of 3 consultations (IQR 1–5) compared with 0 consultations in controls (IQR 0–1)	[18]
0–15	All cancers, Denmark (national), diagnosis 2008–2015 75.3% of children with cancer consulted the general practice within 3 months before diagnosis Socioeconomic gradient in frequency of consultations with highest odds of frequent use of consultations among children from low income (odds ratio 1.94 (95% CI 1.24–3.03) compared to high income families Consultations increased significantly from 16 to 18 months before diagnosis, with a progressive rise from 10 to 12 months before, especially in the last 3 months	[30]
0–25	Average number of health-care provider (HCP) visits before diagnosis of a brain tumour in patients that did not have a tumor predisposition syndrome was 2.4	[32]
0–14	All cancers, England (national GP research database, 600 practices) diagnosis 1988–2010 Median number of consultations in the 3 months before diagnosis: 3 (IQR 2–4) compared with 1 (IQR 1–2) in controls 35.5% had ≥ 4 consultations compared with 9.1% of controls	[19]
0–24	Brain tumours. UK. Diagnosis 1989–2006 Presentation rate to hospital increased from 1.3 per 100 person-months (95% CI 1.1 to 1.4) 6–12 months before diagnosis to 6.4 per 100 person-months (5.8 to 7.0) 1–3 months before, rising steeply to 134.0 (130.4 to 137.6) in the final month before diagnosis. Corresponding rates for presentation to primary care were 8.4 (95% CI 6.8–10.5), 26.7 (21.7–32.8), 148.9 (131.9–168.1) The proportion of emergency presentations to hospitals for children with brain tumours rose steadily from 35% over 12 months before diagnosis to 55% by the time of diagnosis Patients were seen in primary care over four times as often as in hospital up to the final month before the tumour was diagnosed	[26]
15–39 yrs	All cancers, Denmark (national), diagnosed 2002–2011 A progressive increase in consultations was observed for teenage and young adult cases, especially during the last 3 months before cancer diagnosis	[22]
0–24 yrs	Brain tumours, UK, diagnosed 1989–2006 Consultation rates in primary care peaked in the final month before diagnosis at rates over 100 consultations per 100 person months Hospital admissions were most frequent within 1 month before diagnosis with rates over 100 admissions per 100 person months	[27]
0–15 yrs	All cancers, Denmark, diagnosed 1998–2016 The number of contacts with health care was particularly high during the last 3 months before diagnosis, 47% of children showed frequent contacts (≥ 8), and 43% had frequent emergency contacts (≥ 2)	[36]
0–18 yrs	All cancers, UK, presentation 2020–2023 Majority (74%) had 1–3 pre-referral consultations Majority (74%) had 1–3 pre-referral consultations	[41]
0–39 yrs	Sarcomas, Denmark (National), diagnosed 1997–2020 Sarcoma patients more likely than the background Danish population to have contacts in the primary healthcare sector, hospital outpatient clinics, and emergency rooms, and to fill prescriptions for pain- and antimicrobial medication for up to 24 consecutive months before diagnosis	[40]

Measures of variability (median and range, standard deviation (SD), interquartile range (IQR), or 95% CI) are reported as provided in the source publication; where a source publication did not report a variability measure, none is shown. Additional information is available in the appendix (Online resource 4) and the cited reference

*CNS* central nervous system

**Table 3**

- **Published form:** Table formatting deviates from the submitted version.

**Table 3:** Intensity of child health surveillance and usual first contact health care practitioner by country (BENCHISTA Project participants)

	Number of full physical examinations within routine child health practices freely provided for children <5 years*	Intensity of child health surveillance*	Usual healthcare practitioner providing initial assessment of symptomatic young child
Australia	2	Low	GP
Ireland	2	Low	GP
UK	2	Low	GP
Norway	4	Low	GP
Sweden	4	Low	GP
Japan	5	Medium	Paediatrician
Malta	5	Medium	GP
Denmark	7	Medium	GP
Slovenia	8	Medium	Paediatrician
Hungary	9	Medium	Paediatrician
Switzerland	9	Medium	Paediatrician
Austria	10	High	Paediatrician
Estonia	10	High	GP
Germany	10	High	Paediatrician
Greece	10	High	Paediatrician
Poland	10	High	Paediatrician
Spain	10	High	Paediatrician
Canada	11	High	GP or community paediatrician or nurse; varies in urban vs rural settings
Romania	11	High	GP
Belgium	12	High	Health-care nurse or paediatrician
Brazil	12	High	GP
Czech Republic	12	High	Paediatrician
France	14	High	Paediatrician
Portugal	14	High	Youth health-care nurses followed by youth doctors
Italy	15	High	Paediatrician
Bulgaria	21	High	GP

*Classification of countries as low (< 5), medium (5–9), or high (≥ 10) is based on the number of universally offered child health checks that include a full physical examination conducted by a doctor (not nurse) and was assigned by the study authors

*GP* general practitioner

- **Corrected form:** Table 3 has been reformatted to centrally justify the number of child health checks.

**Table 3:** Intensity of child health surveillance and usual first contact health care practitioner by country (BENCHISTA Project participants)

	Number of full physical examinations within routine child health practices freely provided for children <5 years*	Intensity of child health surveillance*	Usual healthcare practitioner providing initial assessment of symptomatic young child
Australia	2	Low	GP
Ireland	2	Low	GP
UK	2	Low	GP
Norway	4	Low	GP
Sweden	4	Low	GP
Japan	5	Medium	Paediatrician
Malta	5	Medium	GP
Denmark	7	Medium	GP
Slovenia	8	Medium	Paediatrician
Hungary	9	Medium	Paediatrician
Switzerland	9	Medium	Paediatrician
Austria	10	High	Paediatrician
Estonia	10	High	GP
Germany	10	High	Paediatrician
Greece	10	High	Paediatrician
Poland	10	High	Paediatrician
Spain	10	High	Paediatrician
Canada	11	High	GP or community paediatrician or nurse; varies in urban vs rural settings
Romania	11	High	GP
Belgium	12	High	Health-care nurse or paediatrician
Brazil	12	High	GP
Czech Republic	12	High	Paediatrician
France	14	High	Paediatrician
Portugal	14	High	Youth health-care nurses followed by youth doctors
Italy	15	High	Paediatrician
Bulgaria	21	High	GP

*Classification of countries as low (< 5), medium (5–9), or high (≥ 10) is based on the number of universally offered child health checks that include a full physical examination conducted by a doctor (not nurse) and was assigned by the study authors

*GP* general practitioner

**Acknowledgements**

- **Published form:** Madhu
- **Corrected form:** Madhumita

**Consortium Names and Affiliations**

- **Published form:** AnnaGavin (affiliation 92)
- **Corrected form:** Anna Gavin
- **Published form:** The section heading "Clinical expert leads" and four clinical expert representatives were omitted following Meric Klein (17).
- **Corrected form:** A new section heading "Clinical expert leads" has been added after Meric Klein (17) to include: Simon Bailey^99^, Nathalie Gaspar^100^, Filippo Spreafico^101^,^102^, Sandra Strauss^103^.
- **Published form:** The respective institutions for the four new clinical expert leads were omitted from the end of the affiliation list.
- **Corrected form:** The following affiliations have been added to the bottom of the list after affiliation 98:
  ◦ 99 Sir James Spence Institute, Royal Victoria Infirmary, Newcastle upon Tyne, United Kingdom
  ◦ 100 Department of Oncology for Child and Adolescent, Gustave Roussy Cancer Campus, Villejuif, France.
  ◦ 101 Paediatric Oncology Unit, Fondazione IRCCS Istituto Nazionale dei Tumori, Milan, Italy.
  ◦ 102 Oncology Unit, IRCCS Istituto Giannina Gaslini, Genoa, Italy.
  ◦ 103 Department of Oncology, UCL Cancer Institute, University College London, London, UK.

The original article has been corrected

