# Supplementary material for: International variation in child healthcare practices and implications for childhood cancer diagnosis: a scoping review and cross-country clinician survey
Source: Eur J Pediatr. 2026 Jun 12;185(7):491. doi: 10.1007/s00431-026-07109-9 (PMC13263281; doi:10.1007/s00431-026-07109-9)
Supplement: Supplementary file 1 — (PDF 903 KB) [file 431_2026_7109_MOESM1_ESM.pdf]

Online resource 1 Selection criteria 2
Online resource 2 Search strategy 3
Online resource 3 Clinician survey 4
Online resource 4 Information extracted from the literature review 6
Online resource 5 Flow chart of clinician recruitment to the survey and characteristics of respondents 27
Online resource 6 Routine child health surveillance programmes from countries included in BENCHISTA 30

Online resource 1. Selection criteria

Inclusion Criteria	Exclusion criteria
**Population:** Children and teenagers (healthy and with cancer diagnosis).<br><br>**Keywords included in title and/or abstract:** surveillance/routine check/health check/health practices/screening/primary care/ prevention/childhood practices/acute care/diagnosis<br><br>**Countries included:** countries with cancer registries that participated in the creation of the BENCHISTA protocol*.<br><br>**Outcomes:** cancer suspicion, serious condition, incidence of finding, delayed diagnosis, cancer diagnosis, childhood cancer, serious illness, health-related issues, health outcomes, early diagnosis, cancer (or similar).<br><br>**Practitioners involved:** nurses, doctors, and paediatricians<br><br>**Publication dates:** 01/01/2012–15/10/2025<br><br>**Type of publication:** literature review, review, peer-reviewed article, report, meta-analysis, evidence-based healthcare, systematic review, population-based study.<br><br>**Language:** English and Spanish.	**Population:** Adult population only, exclusively immigrant populations, populations with other comorbidities.<br><br>**Type of publication:** Conference abstracts, case studies, wirefeeds, commentaries, correspondences, posters, dissertations, theses, magazines, newspapers, trade, journals, books, general information, news, conferences, podcasts, blogs, undefined.<br><br>**Area of study:** Countries not included in the BENCHISTA project.<br><br>**Publication period:** Published prior to 01/01/2012.<br><br>**Intervention:** long-term effects, follow-up, monitoring, interventional studies, clinical trials (RCTs), diet-based interventions.<br><br>**Screening:** blood test, imaging, additional surveillance screening for family history of cancer, genetics.

*Countries involved in BENCHISTA concept and protocol development:
Central Europe: Austria, Belgium, France, Germany, the Netherlands, and Switzerland.
Northern Europe: Denmark, Norway, and Sweden.
Eastern Europe: Bulgaria, Czechia, Estonia, Hungary, Poland and Romania.
Southern Europe: Croatia[†], Greece, Italy, Malta, Portugal, Slovenia and Spain.
British Isles: UK (Northern Ireland, England, Scotland, Wales), and Ireland.
Non-European countries: Australia, Brazil, Canada, Japan and the USA[†].

[†]Although participants in the project concept or protocol development, the USA and Croatia ultimately could not submit the necessary data to the BENCHISTA database and did not form part of Phase 1 of the BENCHISTA project. The other 27 countries completed Phase 1.

Online resource 2. Search strategy

Ovid MEDLINE(R)
1 exp Pediatrics/ or exp Child/ 2106159
2 exp Population Surveillance/ 74020
3 exp Public Health Surveillance/ 5169
4 routine health check.mp. 317
5 exp Primary Health Care/ 183159
6 exp Diagnosis/ 9120453
7 exp Neoplasms/ or solid tumour.mp. 3693893
8 cancer.mp. 2019078
9 2 or 3 or 4 or 5 256167
10 7 or 8 4232444
11 1 and 6 and 9 and 10 530
12 limit 11 to yr="2012 -Current" 253

Embase
1 exp pediatrics/ 112392
2 exp child/ 2729633
3 exp population surveillance/ 105
4 exp public health surveillance/ 361
5 routine health check.mp. 464
6 exp primary health care/ 189099
7 exp diagnosis/ 6951387
8 solid tumour.mp. 2985
9 exp neoplasm/ 4837631
10 cancer.mp. 3997511
11 1 or 2 2776055
12 3 or 4 or 5 or 6 190003
13 8 or 9 or 10 5550793
14 7 and 11 and 12 and 13 424
15 limit 14 to yr="2012 -Current" 307

SCOPUS
(TITLE-ABS-KEY (pediatric* OR child*) AND TITLE-ABS-KEY ("population surveillance" OR "public health
surveillance" OR "routine health check" OR "primary health care") AND TITLE-ABSKEY
(diagnos*) AND TITLE-ABS-KEY (neoplas* OR cancer OR "solid tumour")

Web of Science
(pediatric* or child*) and ("population surveillance" or "public health surveillance" or "routine health check") and
(diagnos*) and (neoplas* or cancer or "solid tumour")

ProQuest Central
(pediatric* OR child*) AND ("population surveillance" OR "public health surveillance" OR "routine health check" OR
"primary health care") AND diagnos* AND (neoplas* OR cancer OR "solid tumour")
Exclude wire feeds
Peer reviewed only
Only articles, literature reviews or reviews
English, Spanish

Online resource 3. Clinician survey

1. In which country are you currently practising?

2. What is your current role?

   a. Family doctor / general practitioner

   b. Paediatrician working in the community

   c. Paediatrician working in hospital

   d. Paediatrician working in both hospital and the community

   e. Other, please specify

3. Does the training to become a Family Doctor/General Practitioner/General Doctor in your country include mandatory paediatric training after basic qualification as a doctor?

   Yes/No

4. Is there a professional organisation (paediatric/child health college) specific to doctors training/trained in paediatrics in your country?

   Yes/No

5. If you answered YES in question number 4, please provide the name of the professional organisation and relevant web link(s).

6. Is there a national mandatory routine child health check (surveillance) programme in your country?

   Yes/No

7. If you answered YES in question number 6, please provide the programme's name and relevant web link(s).

8. Which practitioners are involved in performing routine child health checks (growth and development assessment with physical examination as required)?

   a. Family doctor / general practitioner

   b. Paediatrician working in the community

   c. Paediatrician working in hospital

   d. Paediatrician working in both hospital and the community

   e. Other, please specify

9. If the check includes physical examination, who performs it?

   a. Family doctor / general practitioner

   b. Paediatrician working in the community

   c. Paediatrician working in hospital

   d. Paediatrician working in both hospital and the community

   e. Other, please specify

For questions 10 to 13, please exclude immunisation visits where physical examination is not done.

10. How many routine child health checks are done within the first year of life? *If not known/unsure of exact number, please leave field blank

11. Following question 10, how many of these checks involve physical examination of the whole child, including the abdomen? *If not known/unsure of exact number, please leave field blank

12. How many routine child health checks are done between the ages of 1 to 5 years old? *If not known/unsure of exact number, please leave field blank

13. Following question 12, how many of these checks involve physical examination of the whole child, including the abdomen? *If not known/unsure of exact number, please leave field blank

14. At what age does the routine child health check programme end? Upper age limit: *If not known/unsure of exact number, please leave field blank

15. Is a written list of alarm signs/symptoms of serious diseases in childhood made available to parents/guardians as part of the routine health check programme to indicate when to seek further medical

attention?

Yes/No/Not routinely/Do not know/unsure

16. Who would be the usual first healthcare provider for a parent seeking medical advice/assessment when they are worried their 5-year-old child might be unwell.

a. Family Doctor/General Practitioner

b. Community-based paediatrician (office paediatrician)

c. Community-based nurse

d. Hospital Emergency Department

e. Hospital paediatrician/Paediatric clinic

f. Other, please specify

17. What is the upper age threshold (in years) for accessing a paediatric consultation in your country? Upper age limit: *If not known/unsure of exact number, please leave field blank

18. Would you be happy to be contacted for a follow-up interview after the completion of this survey?

Online resource 4. Information extracted from the literature review

Ref.	Title	Data collection period	Source of data & country	Primary outcomes	Secondary outcomes	Routes or time to diagnosis detailed in paper	Alarm signs/symptoms detailed in paper	Factors affecting early diagnosis	Factors impacting delayed diagnosis	Type of practitioners involved in study	Limitations	Conflicts of interest
**[19]**	Features of childhood cancer in primary care: A population-based nested case-control study	1 Jan 1988 to 31 Dec 2010	General Practice Research Database (GPRD)<br><br>UK	Risk of cancer in children with alert symptoms in the 12 and 3 months before diagnosis, with comparison between cases and controls. Additionally, consultation frequency in the 3 months before diagnosis was recorded. Positive predictive values for having cancer based on alert symptoms and consultation patterns.	Not specifically mentioned.	27% of patients had symptoms in the 3 months before diagnosis. Having symptoms increased odds of cancer up to 28.8-fold compared to a consult without symptoms. Displaying alert symptoms increases the probability of diagnosis. Median consultation rates in the 3 months before diagnosis were three consultations (35.5% had 4+ consultations).	Alert symptoms and frequent consultations are associated with childhood cancer. Having any alert symptom does alter the prior probability of cancer in the subsequent 3 months.	A lack of knowledge on how relevant some specific symptoms are in primary care can have an impact on the diagnostic pathway. Lack of identification of increased consultation frequency.	Perceived delays in diagnosing childhood cancer can have important implications on the acceptance of diagnosis by patients and families, which can also influence subsequent healthcare-seeking behaviour. GPs' awareness and identification of other clues, including consultation frequency, abnormal examination, multiple symptoms, etc. Potential under-recording in GPRD.	General practitioners (GPs)	Under-ascertainment of some cancers in the 0-4 age group might have influenced the magnitude of associations observed. The data were collected prospectively, precluding recall bias. Recording bias is also possible. Parental concern was not studied.	No
**[21]**	Presenting symptoms of children with cancer: A primary-care population-based study	1 Jan 2007 to 31 Dec 2010	Danish Registry of Childhood Cancer (DCCR), linked with the Danish Civil Registration System and the Danish National Health Service Registry<br><br>Denmark	Presenting symptoms and general practitioners' (GPs) interpretation of symptoms of children with cancer. Symptoms were classified according to the International Classification of Primary Care (ICPC)-2.	Not specifically mentioned	GPs were involved in the diagnostic pathways of 80.3% of children, of whom 41.1% were aged 0–4 years at diagnosis, 30.4% were 5–9 years, and 28.5% were 10–14 years. GPs were most frequently involved in the diagnostic	GPs reported 2.4 symptoms per child. Monosymptomatic cases were reported in 38.3% of children, whereas two or more symptoms were reported in 60.5% of children. Clinical symptoms at first presentation were absent in three patients (1.2%). Symptoms were few and mostly fell into the category of 'general and	Symptoms were few per child, mostly falling into the 'general and unspecified' category, impacting early diagnosis. GPs interpreted symptoms as alarm, serious, or vague, with only 20% presenting alarm symptoms. Interpretation of symptoms varied significantly by diagnosis,	GPs' interpretation of symptoms and variation depending on the diagnosis, indicating a potential impact on the time from symptom presentation to final diagnosis.	GPs	Retrospective nature of the questionnaire-based study makes it prone to recall bias. Other sources of bias might pertain to GPs' actual symptoms interpretations. Potential hindsight bias as GPs knew the child's diagnosis at the time of filling out the questionnaire. General practice was not involved in	No

						workup of children subsequently diagnosed with bone tumours (86.4%).	unspecified,' except for patients with CNS tumours. Headache was reported in 13% and was the most frequently reported neurological symptom.	influencing the time to final diagnosis.			the diagnostic pathway of 62 children (who might potentially have more aggressive tumours or more pronounced symptoms than children attending general practice).	
**[18]**	Features of cancer in teenagers and young adults in primary care: a population-based nested case-control study	1 Jan 1988 to 31 Dec 2010	General Practitioner (GP) practices contributing to the Clinical Practice Research Datalink (CPRD)<br><br>UK	Identification and quantification of symptoms of cancer in teenagers and young adults (TYA) presenting to primary care.	Consultation frequency in the 12 months before diagnosis among cases and controls.	In the 12 months before diagnosis, cases had a median of five consultations compared with 2 in the controls. Differences in consultation rates were most apparent in the 3 months before diagnosis (cases had a median of 3 consultations compared with no consultations in controls). Among cases, 86.9% saw their GP in the 3 months before cancer diagnosis compared with 38.8% of controls. Of these, 43% of cases had consulted four times or more.	Symptoms analysis was limited to leukaemia, lymphoma, CNS tumours and bone/soft tissue sarcoma. Lump/mass/swelling of the head and neck had the highest positive predictive value (PPV). For CNS, seizure had the highest PPV.	Increased consultation frequency could be a factor affecting early diagnosis.	Repeated visits to primary care before referral for investigation could contribute to delayed diagnosis. Non-specific symptoms in teenagers and young adults (TYA) population may be mistaken for common illnesses, potentially leading to delayed diagnosis. Limitations in primary care records, where GPs tend to record diagnoses rather than unexplained symptoms.	GPs	Potential for under-recording due to clinicians' preference to record diagnosis versus symptoms. Small sample size due to the rarity of the disease.	No
**[23]**	Use of Primary Care during the Year before Childhood Cancer	1 Jan 2002 to 31 Dec 2008	The Danish Cancer Registry (DCR), the Danish Civil Registration	Utilisation of health care services as a proxy for early symptoms of	Utilisation of out-of-hours (OOH) contacts and diagnostic procedures during	During the year before diagnosis, childhood cancer (CC)	Not specifically mentioned. The study highlighted the importance of	Presence of non-specific symptoms, symptoms that do not indicate	Knowledge about symptom presentation and adequate identification.	GPs	Potential residual confounding by other factors beyond age and gender matching	No

	Diagnosis: A Nationwide Population-Based Matched Comparative Study		System (CRS), and the Danish National Health Insurance Service Registry (NHSR).<br><br>Denmark	childhood cancer (rates of consultations and diagnostic tests in primary health care)	the year preceding the cancer diagnosis	patients had a higher monthly rate of daytime consultations in primary care than controls.<br>A total of 93.3% of the CC patients and 79.7% of the controls consulted the GP within the preceding year, and 81.8% of the former and 44.8% of the latter had consulted the GP within three months before diagnosis.	recognition of symptoms or signs that increase attendance to health care services.	serious disease but mimic common conditions.<br>Lack of awareness of symptoms and signs that increase attendance to health care services.	Lack of knowledge of children's cancer pathway in general practice.		may still play a role in the results.<br>Lack of information on pre-existing co-morbidities among children could be a limitation.<br>The study did not have data on the reasons for GP encounters or the specific signs observed during consultations, which could impact the interpretation of the results.	
**[20]**	Risk of childhood cancer with symptoms in primary care: a population-based case-control study.	1 Jan 1988 to 31 Dec 2010	General Practice Research Database (GPRD), now called Clinical Practice Research Datalink<br><br>UK	Identification of symptoms and signs in primary care that strongly increase the likelihood of childhood cancer.	Determine positive predictive values (PPV) for specific clinical features.	Focused on primary care.<br>No specific mention of other routes to diagnosis or times to diagnosis.	The study identified 12 symptoms strongly associated with childhood cancer, including pallor, head and neck masses, masses elsewhere, lymphadenopathy, abnormal movement symptoms/signs, and bruising.<br>These symptoms, when combined with multiple consultations within a 3-month period, significantly increased the likelihood of a cancer diagnosis in children.<br>The symptoms with the highest PPVs were pallor, head and neck masses, masses elsewhere, lymphadenopathy, symptoms/signs of	Lack of awareness of key symptoms/signs along with multiple consultations.	Lack of evidence-based guidelines that support primary care research may lead to delays in diagnosis and treatment.<br>Rarty of childhood cancer and challenges to recognise paediatric cancer by GPs.	GPs	Under-recording of symptoms which may impact the accuracy of the data collected.<br>Abdominal masses were only recorded in cases and omitted for further analyses, limiting the understanding of symptoms associated with CC.<br>Some analyses were based on a small sample size.	No

							abnormal movement, and bruising.					
**[24]**	Strategies to accelerate diagnosis of primary brain tumours at the primary-secondary care interface in children and adults.	Study period not explicitly provided.	HeadSmart campaign initiated by a consortium including the Children's Brain Tumour Research Centre, the Royal College of Paediatrics and Child Health, and the Brain Tumour Charity.<br><br>UK	Accelerating the diagnosis of primary brain tumours at the primary-secondary care interface through the implementation of specific strategies and recommendations.	Interval between the first symptom and diagnosis, referral pathways.	The total diagnostic interval varies significantly on an individual case basis, with longer delays associated with multiple referrals between primary and secondary care before diagnosis.	'Alarm symptoms' were found to be rare in primary care cancer patients, with only a fifth of children with cancer having one.<br>The highest positive predictive value (PPV) for brain tumours was for abnormalities of movement, at 0.1%.	Non-specific symptoms.<br>Failure to recognise the seriousness of symptoms or misattributing them to existing conditions.	Several factors such as patient delay, healthcare system factors, sociodemographic, psychosocial, and clinical factors, can influence the speed of diagnosis for cancer, including brain tumours.<br>Older age, male gender, living alone and lower educational level contributing to longer TDIs. Fears about diagnosis of cancer and the consequences, self-medication and nondisclosure of a symptom contribute to patient delay.	Healthcare practitioners (General and paediatric practice)	Study focused on UK context, which might limit generalisability.<br>Did not address the impact of socioeconomic factors on diagnostic delays.	No
**[35]**	A new clinical guideline and national awareness campaign accelerated brain tumour diagnosis in UK children – "Headsmart: Be brain tumour aware"	Jan 2011 to May 2013	Data submission from 18 CCLG treatment centres.<br><br>UK	Measure of the impact of the HeadSmart campaign on the time to diagnosis of paediatric brain tumours.	Enhanced public and professional awareness about childhood brain tumours, measured by HeadSmart materials in clinical consultations, resulting in diagnostic referrals for scanning in primary and secondary care.	The median total diagnostic interval (TDI) was reduced from 9.1 (mean, 25.2) weeks (Jan–June 2011) campaign pre-launch to 6.7 (mean, 21.3) weeks in the second year post-launch.<br>No significant change in patient intervals was observed.<br>Confidence in ability to recognise when	The public had high awareness (identified by >80% of respondents) of warning signs (vomiting, headaches, seizures and vision problems) of brain tumours.<br>And low awareness (identified by <50% of respondents) of warning signs (abnormal head position, lethargy, delayed growth or puberty, excessive thirst, passing a lot of urine) of brain tumours.	No sufficient awareness from patients, family or doctors about the risk or symptoms to confidently request CNS imaging during early symptom development.<br>MRI service availability.<br>Changes in health care service or inequality in health care access.	Lack of public confidence in health systems.<br>Complex diagnostic pathways involving multiple health care professionals.	GPs, emergency medicine practitioners and paediatricians	Lack of control or comparison community due to nationwide approach.<br>Inability to account for all relevant confounding factors like new policies or guidelines.<br>Not able to measure inequalities in health care access with the current dataset.<br>Not feasible to categorise patients according to route to diagnosis as the range of referral routes to CNS imaging is diverse,	No

						a child might have a tumour After launch, rose from 32% to 54% for paediatricians but remained low for GPs, from 11% to 12%					and diagnostic pathways of childhood brain tumours are complex.	
**[25]**	The expanding role of primary care in cancer control	Not explicitly mentioned.	Epidemiological evidence from different population-based studies and other relevant publications in the field of oncology and primary care (i.e., NHS Cancer Plan, Cancer Reform Strategy).<br><br>Multiple countries	Determinants of survival, including public understanding of cancer symptoms, timeliness of diagnosis, screening programme availability, quality of cancer treatments, and the role of primary care in cancer care pathways.	Quality of care in secondary cancer care	Around 85% of cancers are diagnosed after symptomatic presentation to a primary care provider (PCP). For cancers with less distinctive symptoms, such as lung cancer, myeloma, and pancreatic cancer, a third or more patients have three or more PCP consultations before being referred to a specialist.	Around 90% of patients present first with symptoms in primary care.<br>The likelihood of cancer, even with the presence of alarm symptoms, is small in adults and smaller still in children, teenagers, and young adults.<br>The presence or absence of symptoms and signs determines the likelihood of disease, influencing further management decisions by the PCP.	Disease-related barriers (rarity, heterogeneity of cancers and overlap of symptoms with those of common, benign conditions).<br>Patient-related or caregiver-related barriers (inability to articulate symptoms, low health-seeking behaviour, little awareness of cancers).	Low educational attainment of the caregiver has been associated with prolonged diagnostic intervals in children, highlighting the importance of caregiver education and awareness in early diagnosis.<br>For the adolescent group, main barriers related to worry about what the doctor might find (72%), embarrassment (56%), fear (54%), and not feeling confident to talk about symptoms (53%). Low awareness of the features of cancer was also reported among the teenagers, highlighting them as a risk group for delayed diagnosis.	Primary care physicians	Data on symptoms, signs, and diagnostic tests are missing, particularly in patients without a cancer diagnosis.<br>The absence of information on the effectiveness of measures designed to strengthen the coordinating role of GPs is a limitation of the study.	Yes, for three authors, the rest declare no competing interests.
**[26]**	Pattern of symptoms and signs of primary intracranial tumours in children and	Primary care data cohort: 1989 and 2006. Hospital data	National Cancer Registry, CPRD (formerly General Practice Research Database) and HES (Hospital	Age pattern and evolution of symptom/signs of intracranial tumours in both primary and secondary care in	Proportion of emergency presentations to hospitals.	Patients with an intracranial tumour presented with one or more tumour-related symptoms	Primary care: Headache was the most common symptom in patients >5 years. 0-4 years most common symptom was raised		Complexity of patterns of symptom development. Variations in each patient's probability of	Primary care physicians and emergency care physicians.	Authors did not have data on symptoms that occurred before the first clinical contact unless they were reported to general	Yes, for three authors. The rest do not have competing interests.

	young adults: A record linkage study	cohort: 1997 and 2006.	Episode Statistics). UK	children and young adults before diagnosis.		before diagnosis at an overall presentation rate of 3.3 per 100 person-months. The proportion of emergency presentations to hospitals rose steadily from 35% over 12 months before diagnosis to 55% by the time of diagnosis. Presentations in primary care were more frequent than in hospitals. Patients were seen in primary care over four times as often as in hospitals up to the final month before the tumour was diagnosed.	intracranial pressure. Convulsions were uncommon in patients <18 years. Visual disturbances were the most common symptom in 0-4 years. Hospital: a large proportion of admissions with headaches, raised intracranial pressure and convulsions came through emergency.		health care service use can impact availability and completeness of medical records, potentially leading to delays in diagnosis.		practitioners. Potential bias in estimation of the number of visits in patients with slow-growing tumours. Assumed homogeneity in the effect of tumour localisation and morphology	
**[22]**	Primary care use before cancer diagnosis in adolescents and young adults - A nationwide register study.	2002 - 2011	Danish Cancer Registry (DCR), the Danish Civil Registration System (CRS) and the Danish National Health Insurance Service Registry (NHSR)<br><br>Denmark	Consultation rates in primary care.	Diagnostic tests performed in primary care.	A progressive increase in consultations was observed for cases, especially during the last three months before cancer diagnosis. The increase in GP attendance was seen for all cancer types, but it started at different time points depending on	The diagnosis of certain types of cancer, specifically in the group of AYAs, is extremely complex as the 'symptom signature' tends to be unspecific. However, the presence of alarm symptoms or signs is emphasised in the study as crucial for timely cancer diagnosis.	Complexity of cancer diagnosis in AYAs (vague symptoms, low cancer incidence, challenge of distinguishing between malignant and non-malignant illnesses). Awareness in recognising vague symptoms or minor changes.	Complexity of symptoms (vague and uncharacteristic symptoms). AYAs may experience a prolonged diagnostic journey. Variable diagnostic window.	GPs	Cannot exclude that residual confounding by other factors, such as ethnicity, might have played a role. No information on pre-existing comorbidity.	No

						the diagnosis (17m for CNS tumours, 12m for soft tissue sarcomas, 9m for lymphomas, 5-6m for leukaemia, bone tumours and germ cell tumour, and 3m before diagnosis for malignant melanomas).						
**[28]**	Evidence for a delay in diagnosis of Wilms' tumour in the UK compared with Germany: implications for primary care for children.	UK 1988-2011. Germany 1994-2001	SIOP WT 2001 trial and study, IMPORT study.<br><br>Multiple countries	Route to diagnosis and burden of disease at diagnosis for children with renal tumours.	Comparing patient demographics and outcomes for children with Wilms' tumour between the UK and Germany.	Children in the UK are diagnosed with a later stage of disease compared to Western European countries.<br>A higher proportion (27%) of children were diagnosed through screening (10%) or following assessment of non-tumour related symptoms (17%) compared with only 14.7% in the three-centre UK study.<br>Fewer children with Wilms' are picked up in the UK incidentally compared to Germany.<br>Parents often choose to take	Children with symptoms are generally older and have a higher burden of disease. Patients diagnosed following tumour-related symptoms are more likely to have advanced disease requiring more intensive therapy compared to those diagnosed asymptomatically. The time interval between symptom onset and medical attention was short for all groups, usually 1-2 days, showing that delay at this stage is not a major factor.	Failure to pick up relevant symptoms and signs during other healthcare contacts. Discrepancies in routine physical examinations of children between the two countries are also mentioned, with Germany having more structured and frequent health screening protocols compared to the UK.	The UK system of primary care for children, mainly through general practice, may lead to a delay in diagnosing Wilms' tumours compared to direct access to care by paediatricians in Germany.<br>Less routine examinations of children or full physical examination of children with minor symptoms (delay in detection).<br>Less than 50% of GPs who undertake primary care for children have received any formal paediatric training beyond what they get in general practice placements.	UK: GPs. Germany: primary care paediatrician.	Not specifically mentioned.	No

						their child to the emergency room rather than the GP in the UK.						
**[27]**	Where are the opportunities for an earlier diagnosis of primary intracranial tumours in children and young adults?	1989 - 2006	Records from the National Cancer Registry linked to hospital admission records from Hospital Episode Statistics (HES) and primary care consultation records from Clinical Practice Research Datalink (CPRD).<br><br>UK	Identifying patterns of primary care presentations before and after diagnosis of brain tumours in different anatomical locations.	Analysis of hospital visits among children and young adults with different types of brain tumours.	Consultation rates varied between tumour locations after accounting for age and year of diagnosis (P<0.001).<br>Consultation rates in primary care peaked in the final month before diagnosis.<br>The frequency of consultations remained raised 12 months after a brain tumour diagnosis.<br>Hospital admissions were most frequent within one month around the diagnosis.	The commonest reason for consultation or admission was convulsion.<br>The first presentation of a seizure, especially a non-febrile seizure, should be evaluated by a paediatrician or a physician with training and expertise in epilepsy and referred to tertiary service for any doubtful diagnosis or treatment failure.	Tumours with longer presentation history before diagnosis (supratentorial, midline and cranial nerve), common clinical features and lack of awareness or knowledge of potential symptoms or patterns that might indicate a potential cancer diagnosis.	Focal neurological deficits that are uncommon might emerge late in the pre-diagnosis period.	Practitioners in primary and emergency care.	Small number of patients with linked primary care records.<br>Limited population coverage of CPRD (5-10%).<br>The generalisability of findings was impacted due to variations in healthcare use probability.	Yes<br>Two authors were funded by the Brain Tumour charity, and one author is a member of the Children with Cancer UK advisory panel.
**[29]**	Children with cancer: a survey on the experience of Italian primary care pediatricians	March - June 2016	Experiences of paediatricians from Friuli Venezia Giulia<br><br>(northeastern area of Italy)	Primary care paediatricians' perception regarding management of paediatric cancer patients.	Relationship between primary care paediatricians and families after a cancer diagnosis.	In 87.6% of cases, the primary care paediatrician saw the child at symptom onset.<br>82.3% of cases (94% of those seen by the paediatrician) were referred to the hospital.<br>12.4% of children were taken directly to the hospital by parents due	The primary care paediatrician understood the severity in 78.7% of cases.<br>Parents understood the severity in 45.1% of cases.<br>Common onset symptoms (47%) included fever, weight loss, weakness, pallor, and pain.	Symptoms leading to diagnosis were often non-specific, making it challenging to identify cancer early.<br>The relationship between the severity of symptoms and the disease outcome did not show a linear correlation with the quality of the relationship	21% of primary care paediatricians reported a total absence of communication with the referral centre, whose efficacy depended more on personal connections rather than on standardised protocols.	Primary care paediatricians	Generalisability (limited to specific geographical area).<br>The sample was represented by a heterogeneous population.<br>Potential recall bias.<br>Due to data collection constraints, the study was unable to compare patient/paediatrician relationships based on diagnosis and treatment duration, which could have	No

						to acute symptoms. Time from symptom onset to cancer diagnosis ranged from a few hours to 1095 days, with a median of 14 days.		between the primary care physician and the family, suggesting other factors influencing early diagnosis.			provided valuable insights.	
**[44]**	Diagnosis of cancer as an emergency: a critical review of current evidence.	Not explicitly mentioned.	Electronic or administrative patient-record datasets.<br><br>Multiple countries	Definitions, frequency, risk factors, patient-group inequalities and potential for preventing a diagnosis of cancer as an emergency presentation.	Recommendations for public health and health-care interventions and research efforts aimed at addressing this under-researched aspect of cancer diagnosis	Patients diagnosed with cancer via emergency care are more likely to have advanced disease. 30% of emergency presentations are generated by direct emergency referral to hospital services by primary-care physicians. Most patients who present with cancer as an emergency have had prior consultations in the 12 months preceding diagnosis. Most patients initially present to non-specialist clinicians. Patients who are very young or very old are more likely to be diagnosed as emergencies. More than half	In an audit conducted in England, investigators assessed if patients who presented as emergencies had previously consulted with red flag symptoms requiring urgent referral for suspected cancer. The audit revealed that 23% of emergency presenters retrospectively met the criteria for such a referral.	Some cancers have minimal symptoms before a main event leading to the emergency presentation. Availability and extent of primary-care testing	No prior contact with the formal health-care system, minimisation of symptoms, conceptual differences in the way people recognise/interpret their symptoms, fears surrounding cancer and its treatment.	Emergency care physicians.	Limited data are available on patients' symptoms and health-care use before emergency presentation, indicating a gap in understanding the pre-diagnostic phase. It focused on population-based evidence, potentially missing out on specific individual-level factors.	No

						(54%) of all patients who present with cancer between 0-14 years of age are diagnosed as emergencies.						
**[45]**	Procedures performed by general practitioners and general internal medicine physicians - a comparison based on routine data from Northern Germany.	First quarter of 2013 and 2015.	The Association of Statutory Health Insurance Physicians (ASHIP) in the Federal State of Schleswig-Holstein<br><br>Northern Germany	Application of specific procedures by German general practitioners and general internal medicine physicians shedding light on disparities in service provision.	Not specifically mentioned	GP focuses on health screening services, especially well-child visits and adolescent health examinations. Most variances in healthcare provision stem from differences in training programmes. In German rural areas, a shortage of paediatricians leads to children's healthcare services being delivered by GPs or GIM physicians.	Not specifically mentioned	Some areas (rural) noted a declining number of GPs. Training emphasising early diagnosis skills might improve detection rates. An increasing number of non-urgent emergency department visits resulted in overstretched emergency facilities with negative effects on the quality and effectiveness of care provision.	Differences in training can impact the timeliness of diagnosis. Variation in the type of patients and performance of specific procedures might affect the accuracy and speed of diagnosis.	General practitioners and general internal medicine physicians	Limited representativeness that might restrict the generalisability of findings to other regions. Patient mix variation between privately insured and statutorily insured patients. Limited data availability as dataset did not cover private patients.	One of the authors is a GP. No other conflicts of interest.
**[30]**	Utilisation of primary care before a childhood cancer diagnosis: do socioeconomic factors matter? A Danish nationwide population-based matched cohort study.	Jan 2008 to Dec 2015.	Danish Civil Registration System, Danish Cancer Register (DCR), Danish National Health Insurance Service Register (NHSR) and Statistics<br><br>Denmark.	Use of primary care two years before a diagnosis of childhood cancer.	Socioeconomic factors and whether they modify the use of consultations and diagnostic tests.	75.3% of children with cancer consulted the general practice within 3 months before diagnosis. 29.4% of cases had diagnostic tests performed in primary care within 3 months before diagnosis.	Not specifically mentioned.	Children with early-stage cancer often present with non-specific and vague symptoms that mimic common conditions like viral infections. Geographical factors such as distance to healthcare facilities could potentially	A prolonged diagnostic interval may occur if the GP does not suspect cancer initially, leading to delays in diagnosis. Patients with low SEP were observed to communicate less actively and receive less information from GPs, which could	General practitioners		No

						Consultations increased significantly from 16 to 18 months before diagnosis, with a progressive rise from 10 to 12 months before, especially in the last 3 months.<br>29% of children with cancer were frequent users of consultations 3 months before diagnosis.<br>There was a progressive increase in diagnostic test rates in the 4–6 months before diagnosis.		influence utilisation and early diagnosis.	contribute to delays in diagnosis.<br>Poor communication or interaction between the GP, child, and parents can contribute to delayed referrals for specialist investigation.<br>The GP's perception of parents as 'worriers' or too sensitive can influence how seriously their concerns are taken, potentially delaying diagnosis.			
**[31]**	Factors related to out-of-hours help-seeking for acute health problems: a survey study using case scenarios	Dec 2015 to Jan 2016	Danish Civil Registration System, the Netherlands (TNS NIPO), and two consumer panels from Switzerland.	Factors influencing intended help-seeking behaviour in out-of-hours care for acute care problems.	Not specifically mentioned.	Women are less inclined to take their child to out-of-hours clinicians compared to men.<br>Lower educated families and refugees seek help sooner than higher education families.<br>Parents with anxiety are less inclined to take their children to out-of-hours care.<br>Parents who perceived	Not specifically mentioned.	Age, gender, education level, ethnicity, and anxiety can affect individuals' perception of symptoms and willingness to seek help.<br>Access to healthcare services, availability of GP and ease to organise childcare can facilitate or hinder individuals from seeking timely medical attention.	Knowledge of parents of childhood diseases can impact medical advice-seeking behaviour.<br>Lack of social support may lead to less contact of out-of-hours care.<br>Cultural differences may play a role in seeking healthcare services.	GPs, out-of-hours providers and emergency care physicians	Use of paper-based case scenarios to measure help-seeking behaviour, which may not fully represent actual behaviour and could introduce social desirability bias.<br>Response rates were acceptable, but there is a potential for selection bias.<br>The questionnaire was only pilot tested in Denmark, which could have impacted the readability and interpretation of questions for Dutch and Swiss participants	No

						difficulties in organising childcare were more inclined to contact out-of-hours care than parents who did not perceive such difficulties. Individuals who had few or more contacts with their GP were more inclined to contact out-of-hours care than individuals who had no contact with their GP.		Awareness, recognition of health issues and decision to seek medical care promptly.				
[32]	Diagnostic delay and morbidity of central nervous system tumours in children and young adults: a pediatric hospital experience.	Jan 2008 to Dec 2017.	Akron Children's Hospital (ACH)<br><br>USA	Diagnostic delays, morbidity and mortality, and specific barriers in the healthcare system.	Occurrence of tumour of treatment-related complications in the patients.	The median time to diagnosis (TDI) was 42 days with a range of 0-5475 days. There was evidence of a significant difference in TDI by tumour location and age.<br>The average number of healthcare visits before diagnosis was 2.4.	The studied institution showed a shorter interval from symptom onset to diagnosis than currently reported in the literature.	Presence of non-specific symptoms that make early diagnosis difficult. Findings suggested that multiple visits may be required before a definitive diagnosis is reached.	Recognition of symptoms by parents and healthcare providers (HCP) is crucial to avoid diagnostic delay.	Healthcare providers (HCP).	High risk of record bias due to the retrospective design.<br>Location bias, considering patients could seek care at multiple institutions.<br>Small sample size.	No
[33]	What do we know about demand, use and outcomes in primary care out-of-hours services? A systematic scoping review	1995 to 2019	Six databases (CINAHL; Medline; PsyARTICLES; PsycINFO; SocINDEX; and Embase)	Patient demand and patterns of use (service, time of use, demographics of users).	Other outcomes associated with primary care out-of-hours services.	Weekend use of out-of-hour (OOH) services exceeded weeknights.<br>The most frequent users were children <5 years.<br>Lower	Fever and gastrointestinal symptoms were the most common in the under-5s population in OOH services.		Increasing age and prior use of OOH services can lead to a delayed escalation in care.<br>The lack of accurate diagnostic coding for conditions presented during	Out-of-hours (OOH) service clinicians.	The study focused on English language papers and health systems similar to the UK, which may have led to other relevant papers being missed.	No

	of international literature					socioeconomics are associated with more frequent OOH health service use. Cancer-related symptoms equated to 2% of consultations.			out-of-hours care contributes to delayed diagnosis.			
**[34]**	Factors associated with the detection of childhood and adolescent cancer in primary health care: A prospective cross-sectional study	Not specifically mentioned.	Municipalities in the northeastern region of Brazil	Knowledge of primary care physicians and nurses about the most common signs and symptoms of paediatric cancers	Effect of professional characteristics on performance in the questionnaire (testing knowledge of the most common signs and symptoms in childhood and adolescent cancer).	Initial care by the professional conducting the consultation impacted the latency period of the disease, with shorter latency when the first visit was at the hospital.<br>Patients attended by paediatricians experienced a shorter delay in diagnosis time, suggesting their heightened awareness of childhood cancer.<br>Primary care plays a vital role in diagnosing childhood cancer, as studies indicate that most patients diagnosed with childhood cancer initially seek care in primary care settings.	Inaccuracy of childhood cancer symptoms, accompanied by the relative rarity of the associated signs and symptoms, tends to make immediate diagnosis more difficult.<br>49% of professionals did not receive academic training with an emphasis on childhood and adolescent cancer. Only 29.4% received information on training in signs and symptoms of childhood and adolescent cancer.<br>The physicians presented greater assertiveness when compared to the nurses.	Lack of academic training focusing on identifying signs and symptoms of cancer in children impacting on early detection and leading to worse prognosis.<br>Insufficient continuous education.<br>The study highlighted that diagnostic errors are more common in primary care settings.<br>Influence of first contact professional, with paediatricians having shorted delays in diagnosis time.<br>System inefficiencies, lacking proper equipment and personnel to make accurate diagnosis.	Health system issues.<br>Level of training and awareness of the first healthcare professional attending the patient.<br>Parental knowledge about signs and symptoms, along with sociodemographic factors can influence the diagnosis process for childhood cancer.<br>Lack of general training among health care professionals.	51 professionals (30 nurses and 21 physicians)	Limited representation of surveyed population as only included professionals working in primary care for at least 6 months, some refused to participate or were not available.<br>Potential response bias due to reliance on self-reported data.<br>Limited generalisability as only focused on a region of Brazil.	No
**[49]**	Early detection of childhood	Oct – Nov 2019	11 primary healthcare	Identification of possibilities and	Difficulties faced by PHC	Not specifically mentioned.	The study highlighted the lack	Lack of specificity in	Clinical manifestation	PHC professionals	Limited sample size, which limits	No

	and adolescent cancer in primary health care: Possibilities and limitations		(PHC) professionals working in three Basic Health Units (Unidades Básicas de Saúde, UBS)<br><br>Brazil	limitations regarding the importance of early detection of childhood and adolescent cancer according to primary health care (PHC) professionals	professionals regarding the early detection of childhood and adolescent cancer.		of specificity of the signs and symptoms of childhood and adolescent cancer as a limitation.<br>Specific signs mentioned included the identification of abnormal masses during physical examinations, changes in oral cavity, and the need for regular clinical evaluations for early detection.	signs and symptoms.<br>Failure to adhere to accessibility for timely identification and intervention.<br>Need for additional training and capacity to enhance skills in recognising and interpreting clinical findings.	similar to other common benign diseases in children, as well as general signs and symptoms with unspecified locations, which can complicate and delay the diagnosis of potential neoplasms.<br>Lack of communication between different levels of health care.<br>Professionals' insufficient knowledge about childhood and adolescent cancer due to limited contact with such cases or lack of continuing education.	working in the UBS selected for the study.	the generalisation of results.	
**[46]**	The frequency, nature and impact of GP-assessed avoidable delays in a population-based cohort of cancer patients.	Sept 2016 to Feb 2017	Data from the English National Cancer Diagnosis Audit (NCDA)<br><br>UK	Frequency, nature and impact of general practitioner (GP) assessed avoidable delays in cancer diagnosis.	Not specifically mentioned.	Avoidable delays occur in about a quarter of cancer diagnoses and, where they do occur, the median diagnostic interval is increased by approximately two months.<br>Also occurs most frequently in the primary care-led investigation phase.<br>About half of the patients who experienced a	Not specifically mentioned.	Multimorbidity is highlighted as a factor associated with greater odds of avoidable delay.	Avoidable delays were attributed to different phases of the diagnostic pathway (13% in pre-consultation, 49% in primary care, and 38% in secondary care).<br>Patient, provider, or healthcare system factors, or their combination, could lead to prolonged intervals in cancer diagnosis, impacting the timeliness and accuracy of the diagnostic process.	GPs	The study period predates the publication of the 2015 NICE guidance, which may have led to changes in clinical practice.<br>The gap between diagnosis and audit might impact the recall of unrecorded information.<br>Insights from patients and secondary care physicians were not within the scope of the NCDA project.	No

						prolonged primary care or diagnostic interval were not deemed by the GP to have had an avoidable delay.						
**[10]**	Cancer diagnosis in Scottish primary care: Results from the National Cancer Diagnosis Audit.	2014	Information Services Division (ISD) Scotland<br><br>UK	Enable an understanding of how Scottish GPs are currently diagnosing cancer.	Comparison with the published English NCDA data concerning route to diagnosis, prolonged diagnostic pathway intervals and avoidable delays in primary care.	87.1% of the sample presented clinically, with 71.5% initially presenting to a GP. Among these, 62.9% visited a GP surgery, 7.1% had a home visit, 1.2% used out-of-hours services, and 0.3% visited other primary care facilities. A smaller proportion, 3.7%, had their initial presentation at Accident and Emergency.<br>Median PCI (primary care interval) was 5 days (IQR 0–23 days), with 11.3% of patients having a PCI longer than 60 days and 7.7% longer than 90 days.<br>Overall, 49% of cases had no investigations initiated in primary care.	There were statistically significant differences in the proportions of diagnostic routes used between the two countries, with Scottish<br>GPs using the urgent suspected cancer route less frequently.<br>The proportion of English diagnoses made via urgent referrals is significantly higher than in Scotland.	Variations in diagnostic pathways between countries can impact early diagnosis, with Scottish GPs using urgent suspected cancer routes less frequently but having easier access to certain diagnostic modalities compared to English counterparts.<br>Adherence to referral guidelines, public education campaigns, and service initiatives can influence primary care practitioners' decisions on cancer suspicion referrals, affecting the timeliness of diagnosis.	Longer PCI and DI can impact cancer outcomes.<br>Remote patients and those with more comorbidities were found to have longer PCI and DI, indicating that geographical location and health conditions can influence diagnostic delays.<br>Potential variations in health system factors (diagnostic routes) could be influencing diagnostic delays.	GPs and hospital clinicians.	Data collection differences might introduce bias.<br>As data were obtained from a single year, generalisability of the results might be impacted.	No

**[36]**	Socioeconomic position and pre-diagnostic health care contacts in children with cancer in Denmark: a nationwide register study	1998 - 2016	Danish Childhood Cancer Registry (DCCR)<br><br>Denmark	Impact of socioeconomic factors on health care utilisation patterns and disease stage in children and teenagers.	Association between selected socioeconomic variables and the odds of advanced stage of disease at diagnosis in children with cancer.	The number of contacts with healthcare was particularly high during the last 3 months before diagnosis across all diagnostics groups.<br>47% of the 3043 children showed frequent contacts (≥8 contacts) to the health care system, and 43% had frequent emergency contacts (≥2 emergency contacts).	Mention that alert symptoms of childhood cancer are relatively uncommon, making it challenging for healthcare professionals to encounter cases in their careers.<br>Due to the rarity of childhood cancer cases, neither GPs nor emergency department professionals may come across such cases frequently.<br>Parents commonly lack experience in recognising the symptoms of childhood cancer.	Socioeconomic position, such as parental education, affiliation to the work market, and depression, can affect the early diagnosis of childhood cancer.	Lack of understanding of the healthcare system.<br>Families with lower socioeconomic status and individuals of non-western origin tend to utilise healthcare services more frequently before a cancer diagnosis, potentially leading to delays in diagnosis.	GPs and hospital-based practitioners	Not adjusting all socioeconomic and health variables together could lead to overlapping effects and potential confounding.	No
**[37]**	How to suspect cancer in Primary Care.	1980 - 2017	Published literature and guidelines include the BRIGHTLIGHT cohort, qualitative studies, NICE and PAHO guidelines.	To evaluate the correlation between the time to diagnosis (TD) of childhood cancer and its impact on survival rates.	Not specifically mentioned.	Focus on reduction of time to diagnosis (TD) and early treatment are key to reduce morbidity and mortality of childhood cancer.<br>TD can be influenced by factors such as the patient's age, type of tumour, and tumour biology.<br>It highlights that high-grade tumours may have a more abrupt onset of symptoms, leading to a	The recognition of alarm symptoms by families and primary care professionals is a priority.<br>Signs such as persistent and progressive headache, neurological signs and symptoms, afebrile seizures, visual disturbances, petechiae, bruising, and signs of bleeding are highlighted as red flags that should prompt urgent referral to a specialised hospital with paediatric haemato-oncology service.	The presentation of symptoms can vary, with some tumours having non-specific clinical signs leading to a longer TD.	Patients' age, where older patients tend to have a greater delayed time to diagnosis.<br>Depending on the type of tumour, TD can also vary.<br>The biology of the tumour is highlighted as a significant factor influencing delayed time to diagnosis and survival in childhood cancer cases.	Primary care physicians	Observational approach rather than clinical or comparative study.	No

						shorter time to diagnosis.						
**[47]**	Improving Health Equity and Reducing Pediatric Cancer Disparities: The Role of the Medical Home	Not specifically mentioned.	General literature.	Disparities and barriers in paediatric cancer care.	Disparities in survivorship care.	Patients who are economically disadvantaged, publicly insured, and not fluent in English tend to present at later stages of the disease.	Not specifically mentioned.	Access-related factors such as diagnostic testing, referrals and time to treatment initiation can impact early diagnosis. Transportation barriers to large treatment centres can be an important limitation to health care access, affecting early diagnosis in vulnerable populations.	Economically disadvantaged, non-fluent in English language and publicly insured patients tend to present at later stages of the disease.	Primary care providers	Limited generalisability due to focusing on specific populations or settings and timeframe of the study.	No
**[50]**	Childhood, teenage and young adult cancer diagnosis during the first wave of the COVID-19 pandemic: A population-based observational cohort study in England.	Jan 2017 to Aug 2020	QResearch Database (V.45)<br><br>UK	Number of cancer diagnoses per month across various age groups.	Incidence rates of diagnosis, intensive care incidents, time-to diagnosis and time-to treatment intervals.	Time to diagnosis did not significantly differ before or after the pandemic. Those diagnosed during the pandemic are more likely to be admitted to ICU before a formal diagnosis. The median time to treatment was 2 days pre-pandemic and 1 day during the pandemic period.	The study highlighted limitations in capturing all associated symptoms and delays in patient intervals due to the lack of recorded information in electronic health records. Also pinpoints the importance of understanding features of CTYA cancers for accurate diagnosis and treatment.	Fall in incident diagnoses for TYA population, potentially as they seek initial medical advice from primary care before a cancer diagnosis, commonly going to referral pathways similar to adults.	Healthcare disruptions may have led to delayed diagnosis. Changes in health-seeking behaviour. Increased odds of cancer-related intensive care admissions during the pandemic suggest potential delays in diagnosis, leading to more severe disease at presentation.	GPs	Limited generalisability of the findings to the broader population. Changes in diagnostic patterns at a population level are not captured. Potential residual confounding and information bias due to misclassification.	Yes. One of the authors is involved with QResearch and has grants from different organisations at the UK level.
**[48]**	Factors that contribute to disparities in time to acute leukaemia	Jun 2017 to Dec 2020	University of California San Francisco (UCSF) School of Medicine in	Structural factors that act as barriers and facilitators to timely diagnosis for paediatric	Recognising non-disease-related factors contributing to delays in	The time interval from disease onset to diagnosis may be a marker of	The timing of symptom onset was widely variable between	Structural factors include financial concerns.	Provider-parent conflict seen when parents' intuition clashed with the	GPs, family medicine and emergency medicine professionals.	Recruitment bias that might limit generalisation. Potential memory recall bias.	No

	diagnosis in young people: an in-depth qualitative interview study		San Francisco, CA, and the Children's Hospital of Philadelphia (CHOP) in Philadelphia, PA.<br><br>USA	patients with acute leukaemia	diagnosis to reduce outcome disparities in paediatric cancer starting from the primary care setting.	access to care and can contribute to additional morbidity during the initial treatment period.	participants, ranging from days to weeks. The reported symptoms of initial presentation were also variable and non-specific, including rash, fever, pallor, bruising, loss of appetite, fatigue, and pain.	Variability in disease presentation. Vague symptoms and difficulties obtaining timely appointments can limit early diagnosis and highlight the need for improved communication and coordination between patients and healthcare providers.	provider's decisions. Delayed laboratory testing. Vague symptoms being attributed to common conditions.		No inter-rater reliability.	
**[39]**	Prospective registration of symptoms and times to diagnosis in children and adolescents with central nervous system tumours: A study of the Swedish Childhood Cancer Registry	Jan 2013 to Dec 2016	Swedish Childhood Cancer Registry<br><br>Sweden	Total diagnostic interval (TDI), patient interval (PI), and diagnostic interval (DI) for paediatric central nervous system tumours.	Identification of factors associated with prolonged diagnostic times for paediatric patients with CNS tumours in Sweden.	Data regarding time and place for first medical consultation were available for 82% of patients. The first assessment was made by a primary care physician in 32.3% of patients. 17.7% were assessed by a specialised physician in an outpatient practice, and 50% were assessed in emergency wards. The median total interval from symptom onset to the start of treatment was 9.9 weeks (mean 29.3 weeks), median TDI of 8.3	Headache and nausea were the most common symptoms among the studied population. The variability of presenting symptoms and the often-non-specific signs and symptoms preceding a diagnosis rendered early detection challenging. Having a combination of different symptoms was associated with shorter diagnostic time intervals.	Age (9-18 years old) was associated with a longer time to diagnosis (median TDI 11 weeks). Isolated initial symptoms are associated with prolonged diagnostic times. Differences in preparedness and experience in interpreting symptoms along with rapid access to neurological imaging could also influence diagnostic time intervals – this considering first assessment at an emergency ward resulted in shorter time to diagnosis. Availability of imaging equipment (MRI) varies between	In this study, patients with specific tumour types and locations (optic pathway glioma spinal cord tumour and midline tumours) had the longest times to diagnosis. Being an adolescent (different parent surveillance in comparison with children). Level of vigilance of healthcare professionals plays a role in the time to diagnosis. Distinct tumour characteristics in different age groups can influence the time to diagnosis (high-grade tumours had the shortest median TDI).	Healthcare professionals at different levels: Primary care doctors, emergency ward staff, specialised outpatient care.	Time-related data were not available for all patients at all measurement points. Time of symptom onset reported by patients/guardians was subjective.	No

						weeks (mean 26.5 weeks) and median PI of 2.6 weeks (mean 10 weeks). Patients with optic pathway glioma, spinal cord tumours, and midline tumours had the longest lead times in terms of total diagnostic intervals (TDI).		regions in Sweden.				
**[11]**	Assessment of the interval to diagnosis in paediatric bone sarcoma	Dec 2004 and Mar 2020	Emergency department or outpatient clinic at the Department of Orthopaedic Surgery and Radiology at Stanford University School of Medicine<br><br>USA	Interval to diagnosis, initial presentation, patients with negative imaging workup at presentation.	Number of encounters with the healthcare system before diagnosis.	The most frequent location of initial presentation was the outpatient paediatrician setting (81%), followed by the emergency department (18%). Average duration of symptoms was 5.4 months (range 0.1-84). The average number of encounters with the healthcare system before the sarcoma diagnosis was 1.9 encounters (range 1-4). Overall, the average time from initial presentation to biopsy-proven diagnosis was	Not specifically mentioned in the paper.	Initial imaging assessed as unremarkable and clinical findings not leading to advanced images – clinical features potentially not specific. Initial avoidance or use of pain medication before the initial presentation. Recall bias in patient reports of symptom onset.	Clinical awareness that there is a subset of patients who do not have the typical characteristics x-ray findings, potentially due to a small tumour volume at initial presentation or not easily detected on plain x-rays; and requiring advanced imaging when there are persisting symptoms. Differences in health care delivery and accessibility across different regions.	Paediatrician and emergency department physician.	Relatively small sample size. Retrospective review is limited by the accuracy of reporting, quality of documentation and consistency in reporting styles. Conducted at one institution, which might limit its generalisability.	No

						19.5 days (range 0-493). 20% of patients with negative radiographic imaging at initial presentation had a statistically significant prolonged interval to diagnosis of 54 days.						
**[38]**	Identifying early symptoms associated with a diagnosis of childhood, adolescent and young adult cancers: a population-based nested case-control study.	Jan 1998 to Dec 2018	QResearch database<br><br>UK	Clinical features (symptoms and signs) associated with a diagnosis of cancer in children, teenagers and young adults attending primary care.	Not specifically mentioned	Not specifically mentioned	The paper details various symptoms associated with specific children, teenage and young adult (CTYA) cancers, identifying known symptoms and new associations.	Timely diagnosis is challenging due to the rareness and diverse nature of CTYA cancers. Non-specific presentation of symptoms at diagnosis. High levels of diagnostic uncertainty among GPs exist even after awareness initiatives such as HEASDSMART.	Limited awareness and screening strategies for CTYA cancers may contribute to delayed diagnosis.	General practices in England	Study with limited power to detect symptom combinations associated with a diagnosis of specific subtypes of cancer. Categorisation made according to ICCC-3, which might limit the interpretation of subtype-specific results. Study unable to capture young people living away from their domicile, which may affect their recorded deprivation level. Possibility of recording bias of symptoms and residual confounding from unaccounted confounders.	One of the authors is an unpaid director of QResearch and an unpaid consultant of Endeavour Predict Ltd. The rest of the authors do not disclose any conflicts of interest.
**[41]**	Quantifying diagnostic intervals and routes to diagnosis for children and young people	Jan 2022 – Dec 2022	CCLG national prospective cohort, UK	Diagnostic intervals, symptom types, healthcare encounters	Emergency presentation, parent-reported experiences	Yes – detailed symptom, diagnostic and total intervals by tumour type and	Yes – e.g. headache, fatigue, pain, vomiting; variation by tumour group	Rapid referral (CNS, acute leukaemia), known cancer predisposition	Non-specific symptoms, multiple consultations, variation in referral routes	GPs, ED clinicians, hospital paediatricians	Limited pre-hospital record linkage; recall bias from parental reports	No

	with cancer in the UK (Childhood Cancer Diagnosis study, CCD): a population-based observational study.					presentation route						
**[40]**	Use of healthcare services and prescription medication prior to sarcoma diagnosis in children, adolescents, and young adults in 1997-2020: a population-based cohort study.	2012–2018	Danish Childhood Cancer Registry, Denmark	Symptom frequency and type by tumour group	Age variation in symptom presentation	No detailed intervals; focused on first symptoms	Yes – most frequent: headache, fatigue, pain	Symptom specificity (e.g. seizures, eye symptoms)	Non-specific or age-related variation in symptoms	Not specified (registry-based analysis)	Retrospective data; limited to first reported symptom in registry	No

Online resource 5. Flow chart of clinician recruitment to the survey and characteristics (roles) of respondents.

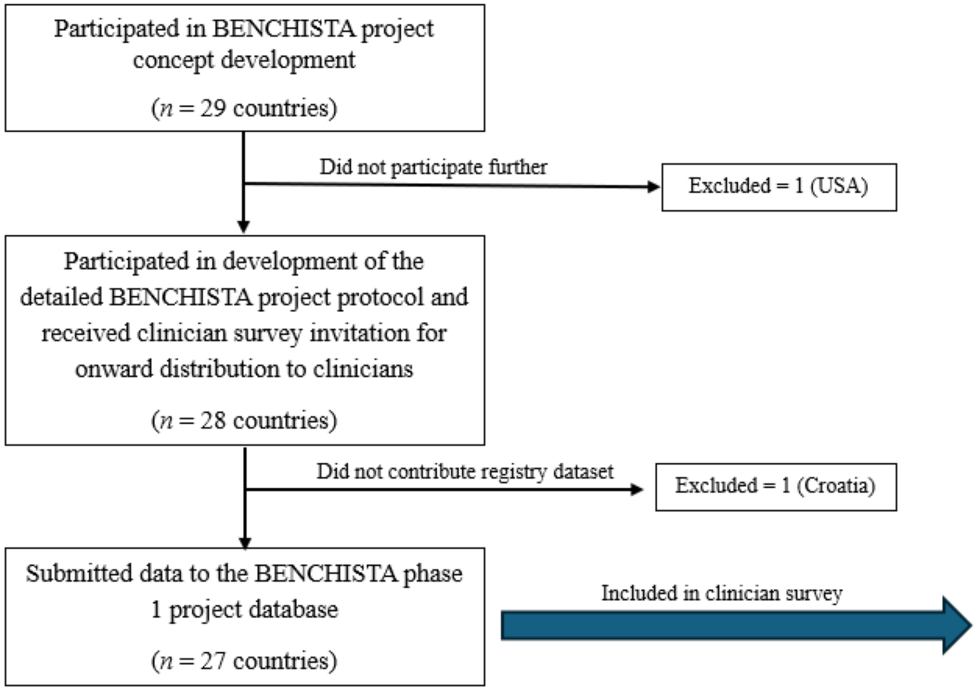

Countries providing clinician response to survey	No. of clinicians responding
Australia	2
Brazil	2
Bulgaria	2
Canada	2
Czech Republic	2
Denmark	1
Estonia	2
Germany	3
Greece	2
Hungary	2
Ireland	2
Italy	2
Japan	2
Malta	2
Norway	2
Poland	2
Portugal	3
Romania	1
Slovenia	2
Spain	2
Sweden	2
Switzerland	2
UK	5
Total = 23 countries	Total = 49 clinicians

For additional information detailing the type of health care professional who responded to the survey, see table on next pages.

Country	Role	Other (specification)
1. Australia	Paediatrician working in both hospital and the community	..
2. Australia	Family Doctor/General Practitioner/General Doctor	..
3. Brazil	Paediatrician working in hospital	..
4. Brazil	Family Doctor/General Practitioner/General Doctor	..
5. Bulgaria	Family Doctor/General Practitioner/General Doctor	..
6. Bulgaria	Family Doctor/General Practitioner/General Doctor	..
7. Canada	Paediatrician working in both hospital and the community	..
8. Canada	Paediatrician working in hospital	..
9. Czech Rep	Family Doctor/General Practitioner/General Doctor	..
10. Czech Rep	Other, please specify	Outpatient emergency care – paediatric haematology and oncology.
11. Denmark	Other, please specify	Neuropaediatrician working in practice
12. England (UK)	Paediatrician working in hospital	..
13. England (UK)	Paediatrician working in hospital	..
14. England (UK)	Paediatrician working in the community (Office paediatrician)	..
15. Estonia	Paediatrician working in both hospital and the community	..
16. Estonia	Family Doctor/General Practitioner/General Doctor	..
17. Germany	Other, please specify	General paediatrician. Current involvement in scientific, paediatric epidemiology
18. Germany	Family Doctor/General Practitioner/General Doctor	..
19. Germany	Paediatrician working in the community (Office paediatrician)	..
20. Greece	Other, please specify	Professor of General Practice and Primary Health Care
21. Greece	Paediatrician working in hospital	..
22. Hungary	Paediatrician working in hospital	..
23. Hungary	Paediatrician working in the community (Office paediatrician)	..
24. Ireland	Other, please specify	Principal Medical Officer, Community Healthcare
25. Ireland	Paediatrician working in hospital	..
26. Italy	Paediatrician working in the community (Office paediatrician)	..
27. Italy	Family Doctor/General Practitioner/General Doctor	..
28. Japan	Family Doctor/General Practitioner/General Doctor	..

29. Japan	Paediatrician working in hospital	..
30. Malta	Family Doctor/General Practitioner/General Doctor	..
31. Malta	Paediatrician working in both hospital and the community	..
32. Northern Ireland (UK)	Paediatrician working in hospital	..
33. Northern Ireland (UK)	Family Doctor/General Practitioner/General Doctor	..
34. Norway	Family Doctor/General Practitioner/General Doctor	..
35. Norway	Paediatrician working in the community (Office paediatrician)	..
36. Poland	Paediatrician working in both hospital and the community	..
37. Poland	Paediatrician working in the community (Office paediatrician)	..
38. Portugal	Family Doctor/General Practitioner/General Doctor	..
39. Portugal	Paediatrician working in both hospital and the community	..
40. Portugal	Family Doctor/General Practitioner/General Doctor	..
41. Romania	Family Doctor/General Practitioner/General Doctor	..
42. Slovenia	Paediatrician working in both hospital and the community	..
43. Slovenia	Family Doctor/General Practitioner/General Doctor	..
44. Spain	Family Doctor/General Practitioner/General Doctor	..
45. Spain	Paediatrician working in both hospital and the community	..
46. Sweden	Paediatrician working in hospital	..
47. Sweden	Family Doctor/General Practitioner/General Doctor	..
48. Switzerland	Paediatrician working in both hospital and the community	..
49. Switzerland	Paediatrician working in the community (Office paediatrician)	..

No responses received from clinicians from four countries who submitted data to the BENCHISTA project (Austria, Belgium, France, The Netherlands). Data for these countries shown in Table 3 and On-line resource 6 were obtained from published data sourced on-line.

Online resource 6. Routine child health surveillance programmes from countries included in BENCHISTA

Country	Professional body specific to paediatric/child health	Routine health surveillance programme	Is it mandatory?	Practitioners involved in providing routine health assessments	Mandatory training required to provide child health checks	Age of Child Health Surveillance Completion (yrs)	Written alarm signs/symptoms*	Reference
Australia	Royal Australasian College of Physicians (RACP). Paediatrics & Child Health Division	Yes<br>Each state provides free routine check-ups for children under its public healthcare system	Not legally mandatory but usually carried out in conjunction with immunisations	General practitioners	No	18	Not routinely	LINK<br>LINK 2
Austria	Austrian Society for Pediatrics and Adolescent Medicine (Österreichische Gesellschaft für Pädiatrie und Jugendmedizin, ÖGP).	Yes<br>Under Mutter-Kind-Pass (Mother-Child Passport) programme	Not legally mandatory but attendance linked to payment of child allowances	Paediatrician and General Practitioners	Yes<br>GPs must meet specific criteria to be approved for conducting the Mutter-Kind-Pass check-ups.	14-16	Not routinely	LINK<br>LINK 2
Belgium	Belgian Society of Pediatrics (Koninklijke Belgische Vereniging over Pediatrie / Société Royale Belge de Pédiatrie)	Yes<br>Le Programme de santé de l'enfant applicable to Flemish and Wallonian region.	Not legally mandatory	Paediatrician	Yes	18	Not routinely	LINK
Brazil	Brazilian Society of Pediatrics (Sociedade Brasileira de Pediatria, SBP)	Yes<br>Known as "Consults de Acompanhamento" which are actual medical check-ups scheduled during childhood.	Yes	General practitioner mainly.	No	10<br>After 10 years old, check-ups become part of adolescent health services.	Yes, general.	LINK
Bulgaria	Bulgarian Society of Pediatrics (Българско дружество по педиатрия).	Yes<br>Program Child Health Care under the Ministry of Health	Yes	General practitioner	No	18	Yes, general.	LINK
Canada	Canadian Pediatrics Society	Each province/territory provides free routine check-ups for children under its public healthcare system (OHIP in Ontario, MSP in British Columbia, RAMQ in Quebec).	Not legally mandatory.	General practitioner	GPs are qualified to perform routine child health check-ups, but their level of expertise differs from that of paediatricians.	18	Not routinely	LINK
Czech Republic	Czech Society of Paediatrics	Yes<br>Under guidelines of the Ministry of Health	Yes	Paediatricians	Yes	19	Yes, general.	LINK

		(Ministerstvo zdravotnictví ČR). These check-ups are part of the Preventive Health Examinations Program (Preventivní prohlídky)						
Denmark	Danish Paediatric Society	Yes<br>Under the Danish Preventive Child Health Programme, which is integrated into the public healthcare system. Programme called Børneundersøgelser (Child Health Examinations)	Not legally mandatory	General practitioners	No	18	Yes, general.	LINK
Estonia	Estonian Paediatric Society<br>(Eesti Lastearstide Selts)	Yes<br>Conducted under the National Child Health Surveillance Program. Laste Tervisekontroll (Child Health Check-Ups)	Not legally mandatory	Family doctors	Yes	19	Yes, general.	LINK
France	Paediatric French Society<br>(Societe Francaise De Pediatrie)	Yes<br>Conducted under the Protection Maternelle et Infantile (PMI) and the Health Booklet (Carnet de Santé) programme.	Certain check-ups in the Carnet de Sante are mandatory.	Paediatricians mainly	Yes	PMI until 6 years old.<br>Carnet de Sante until 18 years old.	Yes, general.	LINK
Germany	German Society for Pediatrics and Adolescent Medicine (Deutsche Gesellschaft für Kinder- und Jugendmedizin e.V. - DG)	Yes<br>Known as "U-Untersuchungen" (U-Checks) and "J-Untersuchungen" (J-Checks) programmes, which are part of the statutory health system.	Early Childhood U-Checks (birth to 5 years) are mandatory in most federal states.<br>J-checks are not legally mandatory	Paediatricians mainly	Yes	U-checks until 6 years old.<br>J-checks until 17 years old.	Yes, general.	LINK<br>LINK2
Greece	Hellenic Pediatric Society	Yes<br>Under the National Health System and follow the Greek Child Health booklet.	Not legally mandatory	Paediatricians mainly	Yes	18	Yes, general.	LINK
Hungary	Hungarian Association of Pediatricians - Young Pediatrician Section	Yes<br>Under the National Health Insurance Fund and part of the Hungarian Child Health Program.	Yes	Paediatricians mainly	Yes	18	Yes, general.	LINK
Ireland	Faculty Paediatrics Royal College Physicians, Ireland.	Yes<br>Under the HSE Child Health & Development	Not legally mandatory.	Public Health Nurses (PHNs) for early development checks.	While a general GP or PHN qualification includes child health	12-13 years	Not routinely	LINK

		Screening Programme, managed by the Health Service Executive (HSE).		General Practitioners for additional child health assessments and vaccinations.	training, further paediatric training or courses may be undertaken for those specialising in child health assessments.			
Italy	Societa Italiana Di Pediatria	Yes<br>Performed through the National Health Service. The programme is known as the "Pediatric check-up program"	Yes	Paediatricians mainly.	Yes	Up to 6 years old. After this age, the patient still receives healthcare services through GPs and paediatricians as needed.	Yes, general.	LINK
Japan	Japan Pediatric Society	Yes<br>Under the Maternal and Child Health System, primarily through the Maternal and Child Health Handbook, the Infant and Child Health Examinations, and the School Health Check-ups.	Not legally mandatory.	Paediatricians as primary providers for early health check-ups.<br>GPs may conduct health check-ups in smaller clinics.	While paediatricians receive dedicated training in child development, GPs also receive basic paediatric education to conduct routine child health check-ups	Maternal and Child Health Handbook (pre-and post-natal care), the Infant and Child Health Examinations (birth to 6 years), and the School Health Check-ups (6–18 years old).	Yes, general.	LINK
Malta	Maltese Paediatric Association	Yes<br>Well Baby and Child Health Screening Programme.	Not legally mandatory.	Paediatricians conduct hospital-based newborn and early childhood screenings.<br>GPs provide primary health care check-ups.	No	Early childhood check-ups (0-5 years).<br>School-age health screenings (5-16 years)<br>Late adolescence (16-18 years, limited surveillance).	Yes, general.	LINK
The Netherlands	Dutch Association for Paediatrics (Nederlandse Vereniging voor Kindergeneeskunde (NVK))	Yes<br>Through the Preventive Child and Youth Healthcare (CYH) service	Not legally mandatory	Paediatricians and GPs	Yes	Up to 18 years	Yes	LINK
Norway	Norwegian Pediatric Association	Yes<br>Conducted under the Child Health Clinic and School Health Service Programme	Not legally mandatory.	Public Health Nurses (primary providers).<br>GPs and Family doctors conduct medical examinations at specific ages.	No	Child Health Clinics (0-5 years old).<br>School Health Services (6-20 years old.	Yes, general.	LINK
Poland	Polish Pediatric Society	Yes<br>Under the Preventive Healthcare for Children	Yes	Neonatal and infant check-ups (0-2 years old) by paediatricians or GPs.	No	18	Yes, general.	LINK

		and Adolescents Programme.		Preschool and school-age check-ups (3-18 years) by primary care doctors.				
Portugal	Sociedade Portuguesa de Pediatria	Yes<br>Under the National Child and Youth Health Programme.	Not legally mandatory.	General practitioners mainly.	No	18	Yes, general.	LINK
Romania	Societe Roumaine De Pediatrie Sociale	Yes<br>Under the National Preventive Healthcare Programme for Children	Yes, for specific ages.	Family doctors mainly	No	18	Yes, general.	LINK
Slovenia	Slovenian Paediatric Society	Yes<br>Under the National Program for the Promotion of Health in Children and Adolescents	Yes, for specific ages.	GPs and family doctors mainly	No	18	Yes, general.	LINK
Spain	Spanish pediatric Association	Yes<br>Under the Child and Adolescent Health Care Program	Yes, for specific ages.	Paediatrician	No	18	Yes, general.	LINK
Sweden	The Swedish Paediatric Society	Yes<br>Under the Child Health Care (BVC - Barnavårdscentral) programme	Mandatory up to the age of 6 years old.	Child health care nurses and family doctors	No	18	Yes, general.	LINK
Switzerland	Professional Society in pediatric and adolescent medicine.	Yes<br>Under the Child and Adolescent Health Program (often referred to as “Kindervorsorge”).	Not legally mandatory.	Paediatricians	No	18	Yes, general.	LINK
UK	Royal College of Paediatrics and Child Health	Yes<br>Healthy child programme	Not legally mandatory	General practitioners	No	Formal routine checks end at 5 years old, but school health services remain available for children and adolescents up to 19.	Not routinely	LINK

Footnote: GPs (General Practitioners). OHIP (Ontario Health Insurance Plan) – The government-funded health insurance programme for residents of Ontario. MSP (Medical Services Plan) – The public health insurance programme for residents of British Columbia. RAMQ (Régie de l’assurance maladie du Québec) – The provincial health insurance programme for residents of Québec. *General alarm signs and symptoms refer to physical and motor development milestones, speech and communication delays, growth and nutrition concerns, and behavioural and social concerns.
